# Trends in prevalence, mortality, and morbidity associated with high systolic blood pressure in Brazil from 1990 to 2017: estimates from the “Global Burden of Disease 2017” (GBD 2017) study

**DOI:** 10.1186/s12963-020-00218-z

**Published:** 2020-09-30

**Authors:** Bruno Ramos Nascimento, Luísa Campos Caldeira Brant, Simon Yadgir, Gláucia Maria Moraes Oliveira, Gregory Roth, Scott Devon Glenn, Meghan Mooney, Mohsen Naghavi, Valéria Maria Azeredo Passos, Bruce Bartholow Duncan, Diego Augusto Santos Silva, Deborah Carvalho Malta, Antonio Luiz Pinho Ribeiro

**Affiliations:** 1 Faculdade de Medicina, Universidade Federal de Minas Gerais, Belo Horizonte, MG Brazil; 2 Hospital das Clínicas, Universidade Federal de Minas Gerais, Avenida Professor Alfredo Balena, 110, Belo Horizonte, MG Brazil; 3 Institute for Health Metrics and Evaluation, University of Washington, Seattle, WA USA; 4 Faculdade de Medicina, Universidade Federal do Rio de Janeiro, Rio de Janeiro, RJ Brazil; 5 Faculdade Ciências Médicas de Minas Gerais, Belo Horizonte, MG Brazil; 6 Programa de Pós-graduação em Epidemiologia e Hospital de Clínicas de Porto Alegre, Universidade Federal do Rio Grande do Sul, Porto Alegre, RS Brazil; 7 Federal University of Santa Catarina, Research Center in Kinanthropometry and Human Performance, Florianópolis, SC Brazil; 8 Escola de Enfermagem, Universidade Federal de Minas Gerais, Belo Horizonte, MG Brazil; 9 Departamento de Vigilância de Doenças e Agravos Não Transmissíveis e Promoção da Saúde, Ministério da Saúde, Brasília, Brazil

**Keywords:** Global burden of disease, Hypertension, Morbidity, Mortality, Epidemiology

## Abstract

**Background:**

Hypertension remains the leading risk factor for cardiovascular disease (CVD) worldwide, and its impact in Brazil should be assessed in order to better address the issue. We aimed to describe trends in prevalence and burden of disease attributable to high systolic blood pressure (HSBP) among Brazilians ≥ 25 years old according to sex and federal units (FU) using the Global Burden of Disease (GBD) 2017 estimates.

**Methods:**

We used the comparative risk assessment developed for the GBD study to estimate trends in attributable deaths and disability-adjusted life-years (DALY), by sex, and FU for HSBP from 1990 to 2017. This study included 14 HSBP-outcome pairs. HSBP was defined as ≥ 140 mmHg for prevalence estimates, and a theoretical minimum risk exposure level (TMREL) of 110–115 mmHg was considered for disease burden. We estimated the portion of deaths and DALYs attributed to HSBP. We also explored the drivers of trends in HSBP burden, as well as the correlation between disease burden and sociodemographic development index (SDI).

**Results:**

In Brazil, the prevalence of HSBP is 18.9% (95% uncertainty intervals [UI] 18.5–19.3%), with an annual 0.4% increase rate, while age-standardized death rates attributable to HSBP decreased from 189.2 (95%UI 168.5–209.2) deaths to 104.8 (95%UI 94.9–114.4) deaths per 100,000 from 1990 to 2017. In spite of that, the total number of deaths attributable to HSBP increased 53.4% and HSBP raised from 3rd to 1st position, as the leading risk factor for deaths during the period. Regarding total DALYs, HSBP raised from 4th in 1990 to 2nd cause in 2017. The main driver of change of HSBP burden is population aging. Across FUs, the reduction in the age-standardized death rates attributable to HSBP correlated with higher SDI.

**Conclusions:**

While HSBP prevalence shows an increasing trend, age-standardized death and DALY rates are decreasing in Brazil, probably as results of successful public policies for CVD secondary prevention and control, but suboptimal control of its determinants. Reduction was more significant in FUs with higher SDI, suggesting that the effect of health policies was heterogeneous. Moreover, HSBP has become the main risk factor for death in Brazil, mainly due to population aging.

## Background

Cardiovascular disease (CVD) is the major cause of morbidity and mortality in developed countries, and the same trend has been observed in Brazil since the epidemiological transition, in the 1960s [1]. Besides being the leading cause of death and hospitalizations in the country, CVD also poses a significant economic burden [1]. Hypertension (HTN) is the most prevalent risk factor for CVD, affecting 32.3% of Brazilian adults [2, 3].

HTN is associated with ischemic heart disease and stroke, the main causes of death in the country, and is also a risk factor for heart failure, chronic kidney disease, cognitive decline, and other diseases [4]. Thus, there is a recognized strong association of HTN with many diseases and early preventive and therapeutic interventions are available. However, the asymptomatic nature of the disease in most of its clinical course and the lack of awareness about the condition are contributing factors for its underdiagnosis, especially in early stages [1]. Published estimates suggest that among hypertensive young adults in the USA, less than 75% are aware of the diagnosis, only about 60% are adequately treated, and around 40% have adequate control [5]. Macinko et al. using data from Brazil National Health Survey conducted in 2013 found that 89% of hypertensive patients had contact with the health system in the past 2 years, but only 65% were aware of their condition, and 33% had their BP under control [6]. In a cohort of Brazilian civil servants, results are similarly suboptimal, with 80.2% awareness among hypertensives and 53% with adequate control [7].

Most of the basic pharmacological arsenal for HTN is freely available in the Brazilian Public Health System (*Sistema Único de Saúde* (SUS)), and national programs, especially in primary care, have been implemented to improve population awareness, early diagnosis, and adherence to HTN treatment [8]. However, the scope of these initiatives is still heterogeneous, and their practical results have not yet been adequately measured. Consistent epidemiological data are needed for the development of health policies in order to reduce the impact of HTN in Brazil.

The primary objective of the present study is to analyze the prevalence of and the burden of diseases attributable to high systolic blood pressure (HSBP) among Brazilians according to sex and federal units (FU) between 1990 and 2017, based on the estimates of the Global Burden of Disease 2017 study (GBD 2017). Additionally, we aimed to assess the correlation between HSBP burden and socioeconomic development.

## Methods

### The Global Burden of Disease study

The GBD study is a multinational research collaboration with the objective to produce consistent estimates of health problems related to more than 328 diseases and injuries in 195 countries and territories. A wide range of data sources (national surveillance—such as the National Health Survey, which measured blood pressure measurements in the Brazilian adult population in 2013 [3]—vital records and verbal autopsy, published and unpublished disease registries, and published scientific literature) and methods were employed to produce specific results by age and sex for the years 1990–2017, updated annually for the entire time series. Estimation techniques imply that Brazilian data, when available, is weighted much more heavily than data from other geographies and primarily drives the estimates. Methods from the GBD 2017 study have been described in detail [9–11]. Methodological specificities of the GBD estimates in Brazil have been previously reported, for the GBD 2015 study [12]. Methods specific to the GBD estimation of HSBP and its burden have been reported for the GBD 2015 study as well [13].

### Methodology for the evaluation of risk factors in the GBD study

GBD’s Comparative Risk Analysis (CRA) is a comprehensive and comparable approach to individual quantification of risk factors, a tool for synthesizing risk evidence and risk-outcome associations. The conceptual CRA framework establishes a causal network of hierarchically organized (5 hierarchical levels) risks or causes that contribute to health outcomes, as well as the attributable burden by age, gender, cause, and location, allowing the quantification of risks or causes at any level of the structure. The relationship between risk exposures and socio-demographic development, as measured by the Sociodemographic Index (SDI), provides additional data on the magnitude of this social interaction [9].

The GBD CRA structure is based on the premise that the risk caused by a given exposure starts at a certain level and then increases as the exposure rises above that level. Four components feed into the calculations to estimate the burden attributable to a given risk-outcome pair: (1) the estimate of the measured burden metric for a cause (i.e., number of deaths, years of life lost (YLLs), years lived with disability (YLDs), or disability-adjusted life-years (DALYs) [10, 11, 14]), (2) the exposure levels for the risk factor, (3) the counterfactual level of risk factor exposure or theoretical minimum risk exposure level (TMREL), and (4) the relative risk of the outcome, related to the TMREL. For a given risk-outcome pair, the attributable DALYs as the total DALYs for outcome multiplied by the Population-Attributable Fraction (PAF) at the risk-outcome pair for a given age, sex, location, and year was estimated. The same applies to the estimation of attributable deaths, YLLs, and YLDs. The PAF is defined as the proportion of burden due to a cause that occurred because of exposure to a given risk factor—considering the counterfactual level of TMREL, in this case for HSBP [9].

For the production of estimates for risk factors, central estimates (means) with 95% uncertainty intervals (95% UIs) were reported. To ensure that UIs capture the uncertainty of all relevant sources (exposures, relative risks (RR), TMRELs, and disease burden estimates), uncertainty was propagated through the analysis. When reported, the percent change estimates were calculated from the central estimates for the time points being compared [9]: the 95% UI was calculated by performing a percent change calculation of the full set of 1000 draws in order to propagate uncertainty. The percent change of the means was then taken to report the mean percent change, and the 95% UI were taken from the draw level calculation.

More detailed methodological information is available in the GBD 2017 publication on risk factor-related burden of disease [9].

### Estimates for high systolic blood pressure

The GBD study estimates for HSBP are produced for individuals ≥ 25 years old. For GBD 2017, 2 new outcomes were added for HSBP: subarachnoid hemorrhage and calcific aortic valve disease, totaling 14 related outcomes (with RRs estimated from published analyses of multiple studies, including a large cohort pooling project [15]): (a) ischemic heart disease, (b) ischemic stroke, (c) hemorrhagic stroke, (d) hypertensive heart disease, (e) cardiomyopathy, (f) atrial fibrillation, (g) aortic aneurysm, (h) rheumatic heart disease, (i) peripheral vascular disease, (j) endocarditis, (k) chronic kidney disease, (l) subarachnoid hemorrhage, (m) calcific aortic valve disease, and (n) other cardiovascular diseases (not listed above). Endocarditis is included due to its association with HSBP in large cohort studies, possibly through the effect of blood pressure on endocarditis-related heart failure. For the outcomes, not only conditions directly resulting from, but also those aggravated by HSBP levels were considered [9, 13]. The aggregate result included death and morbidity. For each outcome, the age- and sex-specific RRs associated with a measured systolic blood pressure were estimated using the DisMod meta-regression tool, considering a TMREL of 110–115 mmHg [9, 13]. For this study, prevalence of HSBP considered exclusively the presence of measured SBP ≥ 140 mmHg, derived from the continuous GBD estimation of SBP, regardless of the diastolic blood pressure (DBP) or the use of anti-hypertensive drugs.

This analysis was divided into 5 components: (1) distribution of HSBP (prevalence) by age, sex, and location; (2) RRs of the 14 outcomes; (3) determining specific PAF for selected outcomes; (4) estimation of attributable deaths and DALYs, stratified by Brazilian federal units, sex, and group of years; and (5) drivers of trends for morbidity and mortality [9–11].

### Metrics of disease burden

The DALYs combine information regarding premature death (YLLs) and disability caused by the condition (YLDs) to provide a summary measure of the healthy years lost due to the condition. The YLLs were calculated by multiplying the deaths observed at each specific age in a certain year by the reference age-specific life expectancy estimated from life table methods. The YLDs were calculated by multiplying health states resulting from sequelae of diseases caused by HSBP (in number of cases/year) by a health-state-specific disability weight representing a degree of lost functional capacity. The process of estimating the burden of the disability has been previously described in detail [10, 11, 14, 16]. The burdens of disability were determined via home interviews in several countries, in which participants were asked to choose between lay descriptions of different health states. For the aggregate of multiple health outcomes attributable to HSBP, adjustment of estimates was performed to account for comorbidity, simulating 40,000 individuals in each age-sex-country-year stratum exposed to the independent likelihood of developing each condition, based on disease prevalence, with 95% UI reported for each estimate [11]. Combining disability weights for each simulated individual, this adjustment adds correlations between coexisting diseases to the model. Age-standardization was obtained via the direct method, applying a global age structure [14, 16] (Supplement 1).

### Drivers of trends

A decomposition analysis of changes in DALYs—a modification of the 3-factor Das Gupta decomposition [9, 17]—was undertaken over the time period into 4 main components, due to changes in (1) population growth, (2) population age structure, (3) risk exposure, and (4) all other factors not included in the analysis, the latter termed as the risk-deleted death and DALY rates. Risk-deleted rates refer to age-standardized death and DALY rates expected if all risk factors included in GBD 2017 were removed, estimated as age-standardized DALY rates multiplied by one minus the PAF for the set of risks. The decomposition analysis was performed for each 5-year time period. The contribution of changes in exposure to the individual risks was scaled to the all-risk effect at the most detailed outcome level [10, 11].

### Sociodemographic index (SDI)

The SDI—weighted geometric mean of *per capita* income, schooling level, and fertility rate under the age of 25—from 1990 to 2017 was used as an estimate of the socioeconomic level of each geographic location, aiming to assess its association with the burden of HTN as a function of the global epidemiological transition [18]. Theoretically, SDI ranges from 0 (lowest) to 1 (highest) and, among countries included in GBD, varies from 0.19 (South Sudan) to 0.94 (Luxembourg).

This study is based on the GBD 2017 data and analytic methods, as described previously in detail [9–11], and on the methodology for data collection and adjustment techniques, as described above. Data were analyzed for the period from 1990 to 2017. All analyses were stratified by sex and presented as absolute and age-standardized estimates for the different FUs of the Brazilian territory, in addition to a comparison of the estimates from Brazil with those of the BRICS countries (Brazil, Russia, India, China, and South Africa) and other countries with universal healthcare (Canada and England), for a better global contextualization of the estimates. Some analyses were further stratified by SDI.

### Ethical considerations

The GBD study was approved by the Institutional Review Board of the University of Washington. There was no need to submit to this research to the local Institutional Review Boards, as the study was conducted in a public domain secondary database, without nominal identification, in accordance with Decree No. 7,724, May 16, 2012, and Resolution 510, of April 7, 2016. The GBD Brazil study was approved by the Institutional Review Board of the Universidade Federal de Minas Gerais, under the protocol CAAE – 62803316.7.0000.5149.

## Results

### Prevalence of HSBP

**Fig. 1:**
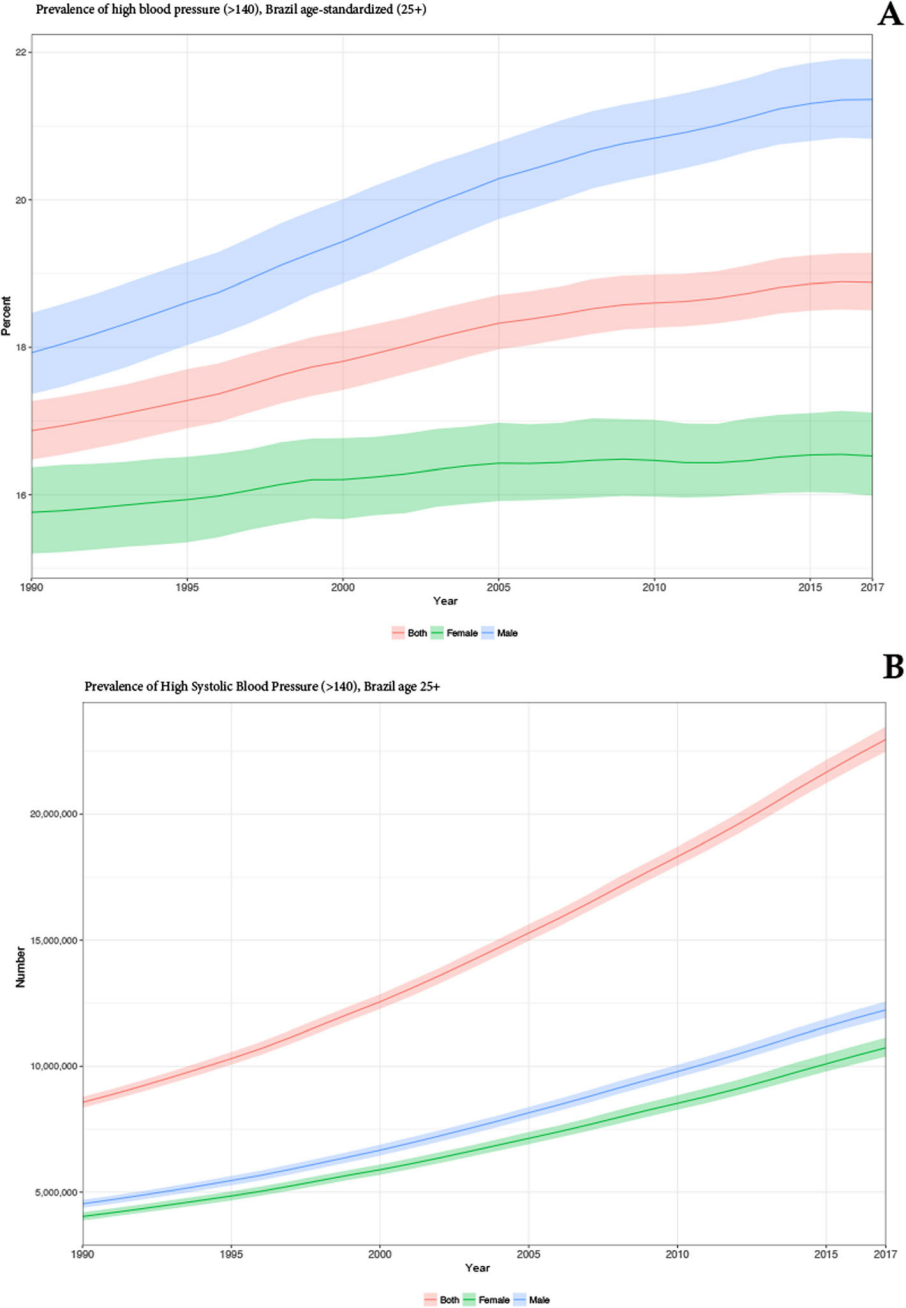
Trends in prevalence of high systolic blood pressure in Brazil for men, women, and both sexes for individuals with ≥ 25 years old, 1990–2017. **a** Age-standardized prevalence. **b** Total number

Table 1 shows the age-standardized prevalence of HSBP in Brazil, which increased from 1990 to 2017, from 16.9% (95%UI 16.5 to 17.3%) to 18.9% (95%UI 18.5 to 19.3%), being higher in men than women along the whole period (Fig. 1a). Although its annualized rate of change is still positive 0.4% (95% UI 0.3 to 0.5%), Fig. 1a reveals that the rise in prevalence rates are declining across the years. If the rates evaluated are not standardized for age, the increase in prevalence is higher (Table 1 and Fig. 1b), due to population aging. Crude and age-standardized prevalence rates and annualized percent change for each federal unit, for both sexes and stratified by sex, are shown in Table 1.

**Table 1:** All ages and age-standardized high systolic blood pressure prevalence in 1990 and 2017, annualized % change in prevalence from 1990 to 2017, for both sexes, for males and females, for individuals with ≥ 25 years old, Brazil and federal units, including the 95% UI

	Male					Female					Both sexes				
	25+		Age standardized (25+)			25+		Age standardized (25+)			25+		Age standardized (25+)		
	1990	2017	1990	2017	Percent change	1990	2017	1990	2017	Percent change	1990	2017	1990	2017	Percent change
**Brazil**	13.9 (13.4 to 14.4)	19.7 (19.2 to 20.2)	17.9 (17.4 to 18.5)	21.4 (20.8 to 21.9)	0.6 (0.5 to 0.8)	11.6 (11.2 to 12.1)	15.8 (15.3 to 16.4)	15.8 (15.2 to 16.4)	16.5 (16 to 17.1)	0.2 (0 to 0.4)	12.7 (12.4 to 13)	17.6 (17.3 to 18)	16.9 (16.5 to 17.3)	18.9 (18.5 to 19.3)	0.4 (0.3 to 0.5)
**Acre**	10.6 (9.3 to 12.8)	14.2 (12.7 to 15.7)	14.6 (13.1 to 16.4)	18.1 (16.4 to 20)	0.8 (0.3 to 1.3)	7 (6.1 to 8)	9.3 (8.2 to 10.5)	12.1 (10.5 to 13.7)	13.2 (11.8 to 14.8)	0.3 (− 0.3 to 1)	8.8 (8 to 10)	11.7 (10.8 to 12.7)	13.4 (12.4 to 14.6)	15.7 (14.6 to 16.9)	0.6 (0.2 to 1)
**Alagoas**	13.2 (11.7 to 15.6)	17.3 (15.5 to 19.1)	16.9 (15.1 to 19)	20.2 (18.3 to 22.1)	0.7 (0.1 to 1.2)	12.7 (11.3 to 14.2)	16.3 (14.6 to 18)	17.5 (15.6 to 19.5)	19 (17.1 to 20.9)	0.3 (− 0.3 to 0.9)	12.9 (11.9 to 14.3)	16.8 (15.6 to 17.9)	17.2 (16 to 18.6)	19.7 (18.3 to 21)	0.5 (0.1 to 0.9)
**Amazonas**	8.8 (7.8 to 9.8)	12.7 (11.4 to 14.5)	14.1 (12.7 to 15.6)	17.2 (15.6 to 19.1)	0.7 (0.2 to 1.3)	5.8 (5 to 6.7)	7.4 (6.5 to 8.5)	10 (8.7 to 11.4)	10.3 (9.1 to 11.7)	0.1 (− 0.6 to 0.8)	7.3 (6.7 to 8)	10.1 (9.2 to 11.1)	12 (11 to 13.1)	13.7 (12.7 to 14.8)	0.5 (0.1 to 0.9)
**Amapá**	8.1 (7.1 to 9.3)	11.5 (10.2 to 13.7)	12.1 (10.7 to 13.5)	14.8 (13.2 to 16.7)	0.8 (0.2 to 1.3)	7.2 (6.3 to 8.3)	9 (7.9 to 10.1)	12.2 (10.6 to 13.9)	13.5 (11.9 to 15)	0.4 (− 0.3 to 1)	7.7 (7 to 8.4)	10.2 (9.4 to 11.3)	12.2 (11.2 to 13.3)	14.2 (13.1 to 15.3)	0.6 (0.1 to 1)
**Bahia**	16 (14.3 to 18.4)	21.4 (19.6 to 23.2)	20.2 (18.2 to 22.3)	24.1 (22.3 to 26)	0.7 (0.2 to 1.1)	12.5 (11.1 to 14.2)	16.5 (14.9 to 18.3)	16.2 (14.3 to 18.2)	17.8 (16.1 to 19.7)	0.4 (− 0.2 to 0.9)	14.2 (13 to 15.6)	18.8 (17.6 to 20.1)	18.1 (16.7 to 19.7)	20.8 (19.5 to 22.2)	0.5 (0.1 to 0.9)
**Ceará**	13.3 (11.8 to 15.6)	17.3 (15.8 to 18.9)	15.4 (13.8 to 17.3)	19 (17.4 to 20.7)	0.8 (0.2 to 1.3)	10.5 (9.1 to 11.9)	13.4 (12 to 14.9)	13 (11.3 to 14.8)	14.5 (12.9 to 16.1)	0.4 (− 0.2 to 1)	11.8 (10.7 to 13)	15.2 (14.2 to 16.3)	14.1 (12.9 to 15.4)	16.6 (15.5 to 17.9)	0.6 (0.2 to 1)
**Distrito Federal**	6.4 (5.6 to 7.3)	11.7 (10.4 to 13.4)	10.3 (9.2 to 11.6)	13.7 (12.3 to 15.4)	1 (0.5 to 1.7)	5.9 (5.1 to 6.8)	10.3 (9.1 to 11.6)	12.4 (10.9 to 14.1)	13.2 (11.9 to 14.8)	0.2 (− 0.4 to 0.9)	6.1 (5.5 to 6.7)	10.9 (10 to 11.8)	11.6 (10.6 to 12.7)	13.7 (12.6 to 14.7)	0.6 (0.2 to 1)
**Espírito Santo**	13.4 (11.8 to 15.7)	19.2 (17.7 to 21)	18.1 (16.3 to 20.1)	21.1 (19.5 to 22.9)	0.6 (0.1 to 1.1)	9.2 (8.1 to 10.5)	13.4 (12 to 15)	13.5 (11.9 to 15.3)	14.2 (12.7 to 15.7)	0.2 (− 0.4 to 0.8)	11.3 (10.3 to 12.5)	16.2 (15.2 to 17.4)	15.8 (14.6 to 17.2)	17.5 (16.4 to 18.7)	0.4 (0 to 0.8)
**Goiás**	12.8 (11.4 to 14.4)	18.7 (16.9 to 20.6)	17.1 (15.5 to 18.9)	20.8 (18.9 to 22.8)	0.7 (0.2 to 1.2)	7.9 (6.8 to 9.2)	11.7 (10.5 to 13.2)	12.6 (11 to 14.4)	13.2 (11.8 to 14.7)	0.2 (− 0.5 to 0.8)	10.4 (9.5 to 11.4)	15.1 (14.1 to 16.3)	14.8 (13.7 to 16.2)	16.9 (15.8 to 18.1)	0.5 (0.1 to 0.8)
**Maranhão**	10.9 (9.8 to 12.1)	14.3 (12.8 to 16.4)	13.8 (12.4 to 15.3)	16.9 (15.3 to 19.1)	0.7 (0.2 to 1.3)	8.7 (7.6 to 9.9)	10.7 (9.4 to 12.2)	11.6 (10.1 to 13.3)	12.8 (11.3 to 14.7)	0.4 (− 0.3 to 1)	9.8 (9 to 10.7)	12.4 (11.4 to 13.6)	12.7 (11.7 to 13.8)	14.8 (13.6 to 16.1)	0.6 (0.2 to 1)
**Minas Gerais**	11.5 (10.3 to 13)	18.8 (16.9 to 20.5)	15.1 (13.8 to 16.7)	19.4 (17.5 to 21.1)	0.9 (0.4 to 1.4)	10.3 (9.2 to 11.4)	15.3 (13.8 to 16.7)	14 (12.5 to 15.4)	15.2 (13.7 to 16.7)	0.3 (− 0.3 to 0.8)	10.9 (10.1 to 11.9)	17 (15.8 to 18.1)	14.6 (13.6 to 15.7)	17.3 (16.1 to 18.4)	0.6 (0.2 to 1)
**Mato Grosso do Sul**	14.2 (12.6 to 15.8)	21.4 (19.5 to 23.5)	18.4 (16.5 to 20.2)	22.9 (20.9 to 25)	0.8 (0.3 to 1.3)	10.6 (9.3 to 12.2)	17 (15.3 to 18.9)	16.8 (14.9 to 19)	18.7 (16.9 to 20.7)	0.4 (− 0.2 to 1)	12.5 (11.4 to 13.6)	19.2 (17.9 to 20.5)	17.6 (16.2 to 19)	20.8 (19.5 to 22.2)	0.6 (0.3 to 1)
**Mato Grosso**	8.9 (7.9 to 10.2)	13.8 (12.2 to 15.8)	12.7 (11.3 to 14.2)	15.4 (13.8 to 17.3)	0.7 (0.1 to 1.3)	6.4 (5.5 to 7.4)	10.3 (9.1 to 11.7)	12.1 (10.6 to 13.8)	12.9 (11.5 to 14.5)	0.2 (− 0.4 to 0.9)	7.8 (7 to 8.6)	12.1 (11 to 13.2)	12.4 (11.4 to 13.6)	14.2 (13 to 15.3)	0.5 (0 to 0.9)
**Pará**	9.1 (8 to 10.8)	12.3 (11 to 14)	12.6 (11.1 to 14.2)	14.9 (13.5 to 16.7)	0.6 (0 to 1.2)	8.6 (7.5 to 9.8)	10.8 (9.7 to 11.9)	13.3 (11.7 to 15.1)	14.2 (12.8 to 15.6)	0.2 (− 0.4 to 0.8)	8.9 (8 to 9.8)	11.5 (10.7 to 12.5)	13.1 (11.9 to 14.2)	14.6 (13.6 to 15.7)	0.4 (0 to 0.8)
**Paraíba**	15.5 (13.9 to 17.2)	18.9 (17.2 to 20.8)	17 (15.3 to 18.9)	20.5 (18.6 to 22.4)	0.7 (0.2 to 1.2)	11 (9.7 to 12.6)	13.2 (11.8 to 14.8)	12.6 (11.1 to 14.4)	13.6 (12.2 to 15.3)	0.3 (− 0.3 to 0.9)	13.1 (12 to 14.3)	15.9 (14.8 to 17)	14.6 (13.5 to 15.9)	16.9 (15.7 to 18.1)	0.5 (0.1 to 0.9)
**Paraná**	15.6 (14 to 17.4)	23.1 (21.1 to 25.1)	20.5 (18.6 to 22.6)	24.4 (22.4 to 26.4)	0.7 (0.2 to 1.1)	12.2 (10.8 to 14)	18.5 (16.7 to 20.4)	17.3 (15.4 to 19.4)	18.7 (16.9 to 20.6)	0.3 (− 0.3 to 0.9)	13.9 (12.8 to 15.1)	20.7 (19.4 to 22.1)	18.9 (17.4 to 20.3)	21.4 (20.2 to 22.8)	0.5 (0.1 to 0.8)
**Pernambuco**	13.9 (12.4 to 16.2)	17.7 (16.1 to 19.4)	16.5 (14.7 to 18.5)	19.6 (17.9 to 21.5)	0.7 (0.2 to 1.2)	11.8 (10.3 to 13.4)	14.7 (13.2 to 16.4)	14.6 (12.7 to 16.4)	15.8 (14.1 to 17.6)	0.3 (− 0.3 to 0.9)	12.8 (11.7 to 14.1)	16.1 (15 to 17.3)	15.5 (14.2 to 16.8)	17.6 (16.4 to 18.9)	0.5 (0.1 to 0.9)
**Piaui**	11.5 (10.2 to 13.5)	15.5 (14.1 to 17)	14.2 (12.7 to 15.9)	16.9 (15.4 to 18.5)	0.7 (0.1 to 1.2)	8.3 (7.1 to 9.6)	11.4 (10 to 12.9)	11 (9.4 to 12.6)	12.3 (10.8 to 13.9)	0.4 (− 0.3 to 1.1)	9.8 (8.9 to 10.9)	13.3 (12.4 to 14.4)	12.5 (11.4 to 13.6)	14.5 (13.4 to 15.6)	0.5 (0.1 to 1)
**Rio de Janeiro**	15.6 (14 to 17.5)	21.7 (19.8 to 23.5)	18.4 (16.5 to 20.3)	21.8 (20 to 23.6)	0.6 (0.2 to 1.1)	14.3 (12.6 to 16.2)	19.5 (17.6 to 21.5)	17.8 (15.8 to 19.9)	18.6 (16.8 to 20.5)	0.2 (− 0.3 to 0.8)	14.9 (13.7 to 16.3)	20.5 (19.2 to 21.9)	18.3 (17 to 19.9)	20.4 (19.1 to 21.7)	0.4 (0 to 0.8)
**Rio Grande do Norte**	13.2 (11.8 to 14.8)	16.9 (15.2 to 18.9)	15.7 (14 to 17.5)	19 (17.3 to 21.1)	0.7 (0.2 to 1.3)	11.3 (9.9 to 12.8)	14.1 (12.7 to 15.7)	13.9 (12.2 to 15.8)	15.1 (13.6 to 16.9)	0.3 (− 0.3 to 0.9)	12.2 (11.2 to 13.3)	15.4 (14.3 to 16.6)	14.8 (13.6 to 16)	17 (15.8 to 18.3)	0.5 (0.2 to 0.9)
**Rondônia**	8.1 (7.1 to 9.2)	12.7 (11.4 to 14.4)	11.7 (10.5 to 13.2)	14.7 (13.3 to 16.3)	0.8 (0.3 to 1.4)	5.5 (4.7 to 6.5)	10.4 (9.4 to 11.5)	12.2 (10.6 to 13.9)	13.8 (12.5 to 15)	0.4 (− 0.1 to 1)	6.9 (6.3 to 7.6)	11.6 (10.8 to 12.6)	12 (11 to 13.1)	14.2 (13.3 to 15.3)	0.6 (0.2 to 1)
**Roraima**	8 (7 to 9.1)	15 (13.4 to 17.1)	16.2 (14.6 to 17.8)	20.2 (18.3 to 22.2)	0.8 (0.3 to 1.3)	4.5 (3.8 to 5.3)	7.4 (6.4 to 8.4)	10.1 (8.7 to 11.6)	10.9 (9.5 to 12.3)	0.3 (− 0.4 to 1)	6.5 (5.9 to 7.2)	11.3 (10.3 to 12.5)	13.6 (12.4 to 14.7)	15.7 (14.5 to 17)	0.5 (0.1 to 0.9)
**Rio Grande do Sul**	16.4 (14.9 to 18.2)	25.2 (23.2 to 27.3)	20.7 (18.9 to 22.5)	25.2 (23.2 to 27.2)	0.7 (0.3 to 1.2)	17.6 (16 to 19.4)	20.4 (18.4 to 22.4)	21.9 (20 to 23.9)	18.8 (16.9 to 20.6)	− 0.6 (− 1 to − 0.1)	17 (15.9 to 18.3)	22.7 (21.3 to 24.1)	21.5 (20.3 to 22.9)	21.8 (20.4 to 23.1)	0 (− 0.3 to 0.4)
**Santa Catarina**	21.2 (19 to 23.7)	27.2 (25 to 29.3)	26.6 (24.2 to 29)	28.8 (26.6 to 31)	0.3 (− 0.1 to 0.7)	13.3 (11.8 to 15)	19.1 (17.4 to 21.1)	19 (17 to 21.2)	19.8 (18.1 to 21.8)	0.2 (− 0.4 to 0.7)	17.2 (15.7 to 18.8)	23.1 (21.6 to 24.5)	22.8 (21.1 to 24.5)	24.3 (22.8 to 25.8)	0.2 (− 0.1 to 0.6)
**Sergipe**	14.7 (13.2 to 16.4)	19.8 (18.1 to 21.5)	19 (17.2 to 20.9)	23.2 (21.5 to 24.9)	0.7 (0.3 to 1.2)	13.8 (12 to 15.6)	17.3 (15.5 to 19.2)	17.9 (15.8 to 20)	19.8 (17.7 to 21.9)	0.4 (− 0.2 to 1)	14.2 (13.1 to 15.5)	18.5 (17.3 to 19.7)	18.4 (17 to 19.9)	21.4 (20 to 22.8)	0.6 (0.2 to 0.9)
**São Paulo**	15.8 (14.1 to 17.6)	23 (21.3 to 24.9)	21.4 (19.4 to 23.5)	24.9 (23.1 to 26.8)	0.6 (0.1 to 1)	12.7 (11.2 to 14.4)	18.3 (16.8 to 20.1)	17.5 (15.6 to 19.7)	18.5 (17 to 20.2)	0.2 (− 0.3 to 0.8)	14.2 (13.1 to 15.4)	20.6 (19.4 to 21.8)	19.4 (18 to 20.8)	21.6 (20.4 to 22.9)	0.4 (0.1 to 0.7)
**Tocantins**	7.1 (6.1 to 8)	9.9 (8.6 to 11.6)	9 (7.9 to 10.2)	11.2 (9.8 to 12.8)	0.8 (0.1 to 1.4)	5.6 (4.8 to 6.5)	7.5 (6.4 to 8.5)	8.7 (7.5 to 10.1)	9.5 (8.1 to 10.8)	0.3 (− 0.4 to 1)	6.4 (5.8 to 7)	8.7 (7.8 to 9.7)	8.9 (8.1 to 9.7)	10.3 (9.3 to 11.4)	0.5 (0 to 1)

### Mortality and morbidity attributed to HSBP

**Fig. 2:**
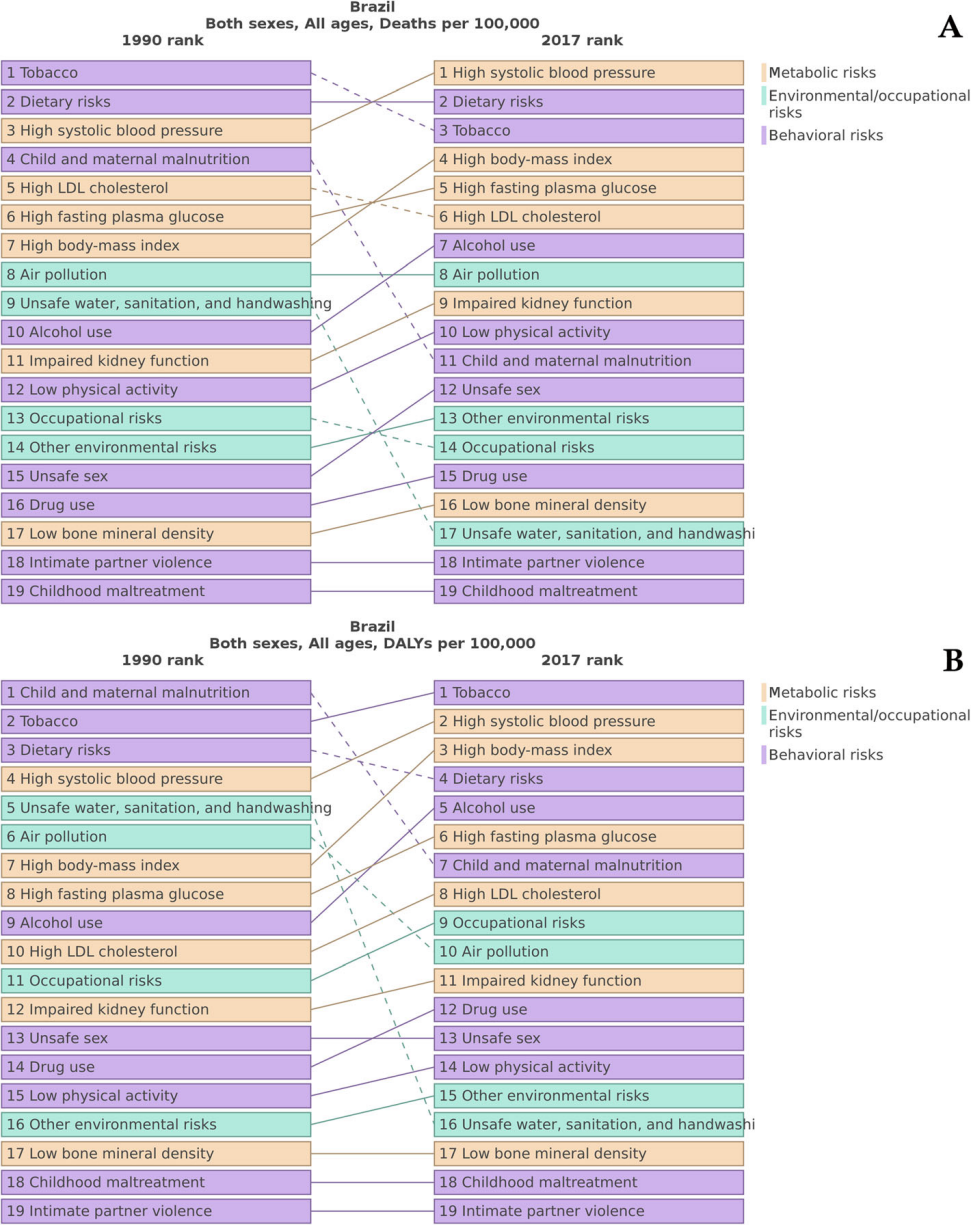
Leading 12 main risk factors for all-causes of death (**a**) and DALY (**b**) in Brazil, for both sexes, in 1990 and 2017

**Fig. 3:**
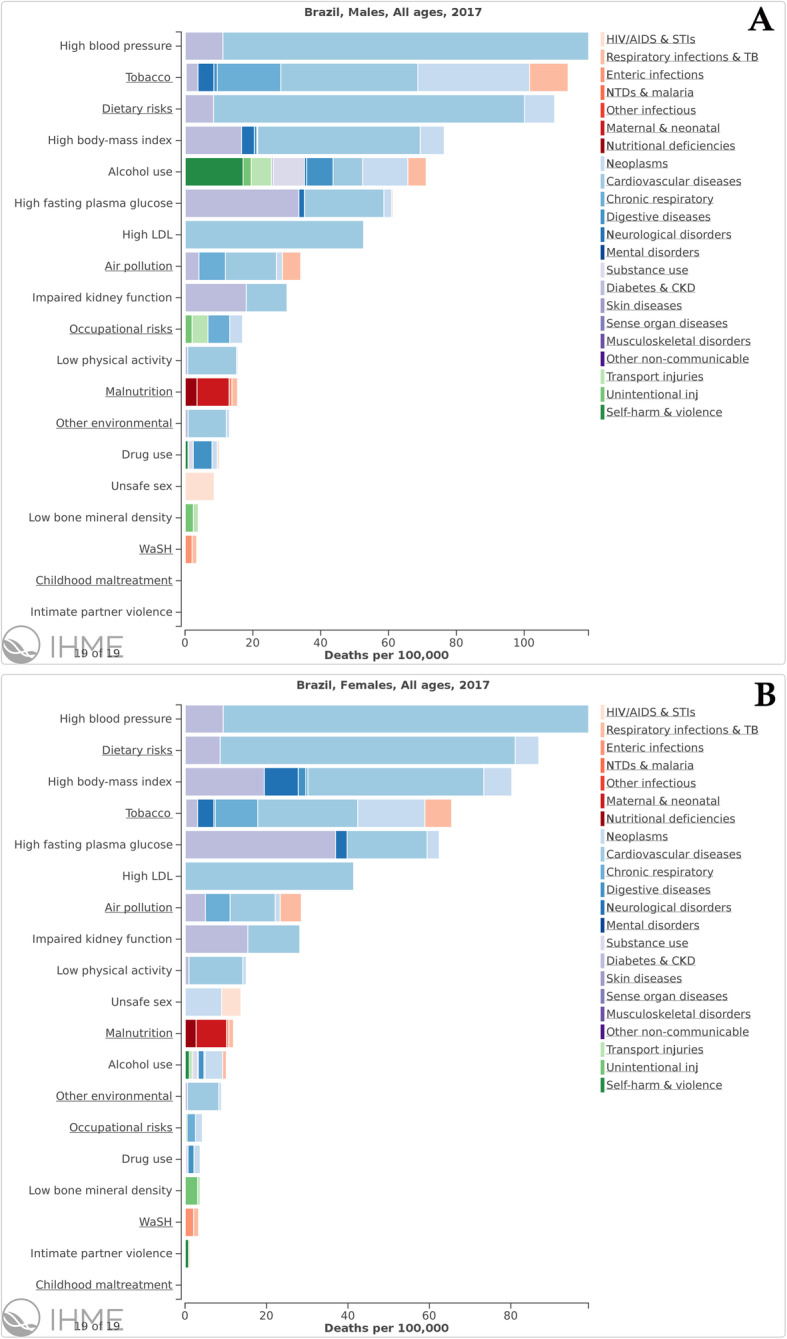
Attributable risk of 17 main risk factors for all-causes of death in Brazil, stratified by sex, in 2017

Figure 2a shows that HSBP is the leading risk factor for the total number of deaths in Brazil and has risen from 3rd in 1990 to 1st position in 2017. During the period, the total number of deaths attributable to HSBP increased 53.4% (95% UI 50.0 to 57.4%) (Table 2), accounting for 150,250 (95% UI 135,714 to 164,122) deaths in 1990 and 230,454 (95% UI 209,698 to 251,499) in 2017. Regarding DALY, HSBP has also gained importance rising from 4th to 2nd position from 1990 to 2017—only behind smoking (Fig. 2b). The leading role of HSBP as a risk factor for death is for both sexes and results from deaths due to cardiovascular and chronic kidney diseases (Fig. 3).

**Table 2:** All age deaths and DALYs, in 1990 and 2017, and percent change of deaths and age-standardized death rates, DALYs, and age-standardized DALY rates between 1990 and 2017 attributable to high blood pressure in Brazil and federal units

	DALYs									Deaths								
	All ages numbers			Crude rates (per 100,000)			Age-standardized rates			All ages numbers			Crude rates (per 100,000)			Age-standardized rates		
	1990	2017	Percent change	1990	2017	Percent change	1990	2017	Percent change	1990	2017	Percent change	1990	2017	Percent change	1990	2017	Percent change
**Brazil**	3,691,929 (3,335,055 to 4,041,905)	5,107,773 (4,642,574 to 5,552,133)	38.3 (34.9 to 42.5)	2470.8 (2232 to 2705.1)	2411.5 (2191.8 to 2621.2)	− 2.4 (− 4.8 to 0.5)	3891.2 (3511.5 to 4261.9)	2223.8 (2022 to 2416.7)	− 42.9 (− 44.1 to − 41.3)	150,250 (135,714 to 164,122)	230,454 (209,698 to 251,499)	53.4 (50 to 57.4)	100.6 (90.8 to 109.8)	108.8 (99 to 118.7)	8.2 (5.8 to 11.1)	189.2 (168.5 to 209.2)	104.8 (94.9 to 114.4)	− 44.6 (− 45.9 to − 42.9)
**Acre**	5239 (4647 to 5853)	12,082 (10,873 to 13,360)	130.6 (115.9 to 145.9)	1258.7 (1116.4 to 1406.3)	1374.3 (1236.8 to 1519.6)	9.2 (2.2 to 16.4)	2897.7 (2577.1 to 3217.9)	1987.7 (1793.2 to 2191.4)	− 31.4 (− 35.4 to − 27.1)	211 (188 to 234)	521 (471 to 575)	146.8 (131.9 to 162.3)	50.7 (45.1 to 56.3)	59.3 (53.6 to 65.5)	16.8 (9.8 to 24.2)	147.6 (129.9 to 165.4)	96.6 (87 to 106.9)	− 34.6 (− 38.5 to − 30.1)
**Alagoas**	52,396 (46,885 to 57,637)	88,644 (80,524 to 96,701)	69.2 (60.4 to 78.4)	2034.7 (1820.7 to 2238.2)	2499.5 (2270.6 to 2726.7)	22.8 (16.5 to 29.6)	3680.9 (3308.1 to 4047)	2826.8 (2568.8 to 3078)	− 23.2 (− 27 to − 19.2)	2246 (2013 to 2472)	3975 (3610 to 4349)	77 (68.4 to 86.1)	87.2 (78.2 to 96)	112.1 (101.8 to 122.6)	28.5 (22.3 to 35.1)	176.6 (157.9 to 195.1)	133.4 (121.1 to 146.2)	− 24.4 (− 28.4 to − 20.5)
**Amazonas**	20,951 (18,531 to 23,377)	47,618 (42,360 to 52,713)	127.3 (113.4 to 143)	1002.8 (887 to 1118.9)	1173.5 (1043.9 to 1299)	17 (9.9 to 25.1)	2503.5 (2228.5 to 2790.7)	1730.1 (1548.4 to 1910.9)	− 30.9 (− 34.8 to − 26.6)	860 (763 to 958)	2073 (1857 to 2291)	140.9 (126.5 to 156.7)	41.2 (36.5 to 45.9)	51.1 (45.8 to 56.5)	24 (16.6 to 32.2)	129.4 (113.3 to 145.8)	85.7 (76.3 to 94.6)	− 33.8 (− 37.6 to − 29.2)
**Amapá**	2596 (2278 to 2916)	9786 (8712 to 10,896)	276.9 (255.8 to 300)	945.3 (829.4 to 1061.7)	1217.8 (1084.2 to 1355.9)	28.8 (21.6 to 36.7)	2424.4 (2142.5 to 2703.4)	1888.9 (1683.2 to 2096.4)	− 22.1 (− 26.2 to − 17.7)	103 (91 to 115)	376 (336 to 416)	265.4 (245.8 to 286.3)	37.4 (33.2 to 41.8)	46.8 (41.8 to 51.7)	24.9 (18.2 to 32)	125.2 (108.9 to 142)	86.9 (77.3 to 96.8)	− 30.6 (− 34.6 to − 25.9)
**Bahia**	225,346 (200,492 to 249,576)	364,453 (331,774 to 397,846)	61.7 (50.8 to 73.4)	1845.8 (1642.2 to 2044.2)	2324.1 (2115.7 to 2537.1)	25.9 (17.4 to 35)	3140.5 (2799.8 to 3468)	2298.7 (2091.8 to 2509.9)	− 26.8 (− 31.5 to − 21.9)	9698 (8623 to 10,657)	16,825 (15,359 to 18,356)	73.5 (62.7 to 85.5)	79.4 (70.6 to 87.3)	107.3 (97.9 to 117.1)	35.1 (26.7 to 44.5)	148.2 (131.6 to 163.2)	105.7 (96.5 to 115.2)	− 28.7 (− 33 to − 23.9)
**Ceará**	88,548 (75,977 to 100,499)	169,274 (150,764 to 187,672)	91.2 (76.6 to 108.9)	1360.9 (1167.7 to 1544.6)	1740.5 (1550.1 to 1929.6)	27.9 (18.1 to 39.8)	2099.3 (1808.4 to 2378.6)	1727 (1541.3 to 1914.5)	− 17.7 (− 23.9 to − 10.3)	3885 (3341 to 4467)	8045 (7078 to 8969)	107.1 (90.8 to 126)	59.7 (51.3 to 68.6)	82.7 (72.8 to 92.2)	38.5 (27.6 to 51.2)	97.3 (83.6 to 112)	81.3 (71.7 to 90.5)	− 16.5 (− 23.3 to − 8.9)
**Distrito Federal**	22,786 (20,013 to 25,471)	40,715 (36,166 to 45,244)	78.7 (67.6 to 93.7)	1408.6 (1237.2 to 1574.6)	1381 (1226.7 to 1534.6)	− 2 (− 8.1 to 6.3)	3317.2 (2935.9 to 3680.9)	1625.1 (1434.9 to 1806.8)	− 51 (− 53.8 to − 47.5)	788 (697 to 871)	1702 (1505 to 1901)	116 (101.6 to 135.4)	48.7 (43.1 to 53.8)	57.7 (51 to 64.5)	18.5 (10.6 to 29.1)	175.9 (153.6 to 197.7)	90.9 (79.6 to 102.1)	− 48.3 (− 51.6 to − 43.6)
**Espírito Santo**	60,740 (54,355 to 66,814)	88,533 (79,953 to 96,596)	45.8 (38.2 to 54.5)	2303.6 (2061.4 to 2533.9)	2273.7 (2053.3 to 2480.7)	− 1.3 (− 6.4 to 4.6)	3994 (3574.6 to 4384.9)	2060.5 (1860.7 to 2247.2)	− 48.4 (− 51 to − 45.5)	2518 (2244 to 2771)	4051 (3677 to 4417)	60.9 (53 to 69.6)	95.5 (85.1 to 105.1)	104 (94.4 to 113.4)	8.9 (3.6 to 14.8)	219.7 (192.5 to 245.8)	99.8 (90.4 to 109.2)	− 54.6 (− 57.1 to − 51.9)
**Goiás**	72,787 (64,876 to 80,732)	137,680 (124,090 to 151,725)	89.2 (79.1 to 100.6)	1779.3 (1585.9 to 1973.5)	2039.6 (1838.2 to 2247.6)	14.6 (8.5 to 21.5)	3324 (2970.4 to 3670.4)	1993.9 (1796.5 to 2195.7)	− 40 (− 43.1 to − 36.8)	2677 (2387 to 2954)	5774 (5207 to 6388)	115.6 (104.6 to 128.4)	65.4 (58.3 to 72.2)	85.5 (77.1 to 94.6)	30.7 (24 to 38.4)	166.2 (145 to 186.5)	92.5 (83.2 to 102.6)	− 44.4 (− 47.3 to − 41.1)
**Maranhão**	88,093 (77,152 to 99,720)	144,681 (128,472 to 160,157)	64.2 (51.4 to 77.4)	1738.3 (1522.5 to 1967.8)	1828.1 (1623.3 to 2023.7)	5.2 (− 3.1 to 13.6)	3149.6 (2773.6 to 3556.4)	2242.8 (1994.2 to 2478.8)	− 28.8 (− 34.1 to − 23.6)	3261 (2856 to 3685)	6440 (5711 to 7117)	97.5 (83.1 to 112.4)	64.3 (56.4 to 72.7)	81.4 (72.2 to 89.9)	26.5 (17.3 to 36)	132 (114.6 to 149.3)	103.8 (92 to 114.8)	− 21.4 (− 26.8 to − 15.7)
**Minas Gerais**	401,227 (355,152 to 444,026)	498,524 (448,406 to 545,161)	24.3 (18.4 to 30.8)	2501.9 (2214.6 to 2768.8)	2343.3 (2107.7 to 2562.5)	− 6.3 (− 10.8 to − 1.4)	3842.9 (3418.8 to 4244.4)	1951.8 (1755.8 to 2134.1)	− 49.2 (− 51.4 to − 46.6)	16,070 (14,305 to 17,664)	22,200 (20,018 to 24,495)	38.1 (32.1 to 44.9)	100.2 (89.2 to 110.1)	104.3 (94.1 to 115.1)	4.1 (− 0.5 to 9.2)	189.8 (166.5 to 212)	88.1 (79.4 to 97.5)	− 53.6 (− 55.8 to − 51)
**Mato Grosso do Sul**	38,218 (34,310 to 42,151)	71,536 (64,998 to 78,333)	87.2 (77.3 to 99.1)	2121 (1904.1 to 2339.2)	2586.4 (2350 to 2832.2)	21.9 (15.5 to 29.7)	3860.7 (3479.4 to 4232.8)	2493.6 (2261.9 to 2724.1)	− 35.4 (− 38.7 to − 31.7)	1458 (1311 to 1603)	3094 (2790 to 3389)	112.2 (101.2 to 124.6)	80.9 (72.7 to 89)	111.9 (100.9 to 122.5)	38.2 (31 to 46.3)	186.5 (164.9 to 208.2)	117.1 (105.4 to 128.8)	− 37.2 (− 40.5 to − 33.5)
**Mato Grosso**	27,107 (23,355 to 30,690)	61,985 (55,391 to 68,518)	128.7 (110.2 to 149)	1357.6 (1169.6 to 1537)	1773.9 (1585.2 to 1960.8)	30.7 (20.1 to 42.3)	3001.2 (2603.9 to 3369.2)	1925.4 (1720.6 to 2123.3)	− 35.8 (− 40.5 to − 31)	986 (851 to 1115)	2560 (2290 to 2821)	159.7 (140.6 to 182)	49.4 (42.6 to 55.9)	73.3 (65.5 to 80.7)	48.4 (37.5 to 61.1)	144.2 (125.1 to 164.9)	92.1 (82.3 to 102.1)	− 36.1 (− 40.9 to − 31.2)
**Pará**	64,966 (57,161 to 72,834)	136,450 (121,642 to 151,330)	110 (96.8 to 124.8)	1322.1 (1163.3 to 1482.2)	1524.5 (1359.1 to 1690.8)	15.3 (8.1 to 23.4)	2870.9 (2536.1 to 3205)	2012.8 (1793 to 2228.1)	− 29.9 (− 34.2 to − 25.2)	2723 (2400 to 3046)	5832 (5194 to 6483)	114.2 (100.6 to 129.4)	55.4 (48.8 to 62)	65.2 (58 to 72.4)	17.6 (10.1 to 25.9)	144.3 (126.5 to 163.2)	94.6 (84.1 to 105.2)	− 34.4 (− 38.7 to − 30.1)
**Paraíba**	65,331 (57,817 to 72,834)	104,999 (93,606 to 116,692)	60.7 (46.8 to 76.1)	1990.1 (1761.2 to 2218.7)	2457.2 (2190.6 to 2730.9)	23.5 (12.8 to 35.3)	2792.8 (2473.7 to 3106.4)	2307.4 (2057.2 to 2561.1)	− 17.4 (− 24.5 to − 9.6)	3041 (2673 to 3402)	5126 (4534 to 5790)	68.6 (54.2 to 85.2)	92.6 (81.4 to 103.6)	120 (106.1 to 135.5)	29.5 (18.5 to 42.3)	135.8 (118.9 to 152.5)	109.8 (97.2 to 123.8)	− 19.1 (− 26.1 to − 11.1)
**Paraná**	234,401 (211,813 to 255,385)	293,998 (267,094 to 320,622)	25.4 (19.5 to 31.8)	2731.8 (2468.5 to 2976.3)	2627.8 (2387.4 to 2865.8)	− 3.8 (− 8.3 to 1.1)	4625.7 (4165.8 to 5051.9)	2276.7 (2068.1 to 2481.4)	− 50.8 (− 52.9 to − 48.4)	9572 (8642 to 10,434)	13,632 (12,366 to 14,927)	42.4 (36.3 to 49.2)	111.6 (100.7 to 121.6)	121.9 (110.5 to 133.4)	9.2 (4.6 to 14.4)	247.6 (217.7 to 274.9)	114.1 (103 to 125.4)	− 53.9 (− 55.8 to − 51.4)
**Pernambuco**	180,635 (162,042 to 198,421)	260,906 (236,375 to 287,057)	44.4 (37.4 to 51.5)	2462.6 (2209.1 to 2705.1)	2619.2 (2372.9 to 2881.7)	6.4 (1.1 to 11.6)	3848.1 (3446.8 to 4227.8)	2605.7 (2361.7 to 2858.2)	− 32.3 (− 35.5 to − 29.1)	7774 (6954 to 8560)	11,647 (10,413 to 12,872)	49.8 (42.7 to 57.1)	106 (94.8 to 116.7)	116.9 (104.5 to 129.2)	10.3 (5.1 to 15.7)	190.8 (169.4 to 213.1)	120.5 (107.6 to 133.3)	− 36.9 (− 40 to − 33.4)
**Piaui**	43,007 (37,685 to 48,236)	75,266 (67,613 to 82,357)	75 (63 to 89.1)	1613.6 (1413.9 to 1809.8)	2102.1 (1888.4 to 2300.2)	30.3 (21.4 to 40.8)	2865.2 (2507.2 to 3205)	2088.8 (1877.5 to 2282.6)	− 27.1 (− 31.9 to − 21.6)	1845 (1615 to 2065)	3617 (3229 to 3984)	96.1 (82.5 to 112.2)	69.2 (60.6 to 77.5)	101 (90.2 to 111.3)	46 (35.9 to 58)	139 (121.7 to 156.2)	100.4 (89.6 to 110.6)	− 27.8 (− 32.5 to − 22.1)
**Rio de Janeiro**	553,453 (499,463 to 606,160)	591,403 (537,174 to 647,548)	6.9 (2.1 to 12.3)	4216.4 (3805.1 to 4617.9)	3397.3 (3085.8 to 3719.8)	− 19.4 (− 23 to − 15.3)	5324.9 (4792.2 to 5841.2)	2701.1 (2448.4 to 2958.1)	− 49.3 (− 51.4 to − 46.9)	21,493 (19,362 to 23,541)	25,956 (23,460 to 28,415)	20.8 (15.4 to 27)	163.7 (147.5 to 179.3)	149.1 (134.8 to 163.2)	− 8.9 (− 13 to − 4.3)	251.9 (223.4 to 280.8)	121.5 (109.5 to 133.2)	− 51.8 (− 53.9 to − 49.3)
**Rio Grande do Norte**	37,770 (33,422 to 42,134)	74,869 (67,367 to 82,559)	98.2 (83.9 to 114.3)	1527.4 (1351.6 to 1703.9)	2060.2 (1853.8 to 2271.8)	34.9 (25.2 to 45.8)	2301.3 (2032.1 to 2562.9)	2004.9 (1802.2 to 2211.1)	− 12.9 (− 19.1 to − 6)	1771 (1543 to 2008)	3539 (3162 to 3943)	99.9 (83.6 to 116.6)	71.6 (62.4 to 81.2)	97.4 (87 to 108.5)	36 (24.9 to 47.4)	112.3 (97.5 to 128)	93.6 (83.8 to 104.2)	− 16.6 (− 23.2 to − 9.5)
**Rondônia**	15,737 (13,723 to 17,908)	31,837 (27,741 to 36,407)	102.3 (77.2 to 128.7)	1413.5 (1232.6 to 1608.4)	1841.1 (1604.2 to 2105.3)	30.3 (14.1 to 47.2)	3702.4 (3253.9 to 4158.8)	2125.5 (1853.1 to 2428.3)	− 42.6 (− 49.1 to − 35.5)	526 (460 to 594)	1353 (1180 to 1546)	157.3 (125.6 to 193.5)	47.2 (41.3 to 53.4)	78.3 (68.2 to 89.4)	65.7 (45.3 to 89)	182.7 (159.6 to 207.5)	104.2 (90.9 to 119.1)	− 42.9 (− 49.1 to − 35.8)
**Roraima**	2323 (1990 to 2676)	7329 (6391 to 8439)	215.5 (171.9 to 264.9)	1106.1 (947.3 to 1274.1)	1297.5 (1131.5 to 1494)	17.3 (1.1 to 35.7)	3525.9 (3076.9 to 4000.7)	2097 (1836.7 to 2408.9)	− 40.5 (− 48 to − 32.2)	79 (69 to 90)	301 (263 to 346)	279.5 (226 to 338.5)	37.7 (32.6 to 42.7)	53.2 (46.5 to 61.3)	41.1 (21.2 to 63)	200.4 (173.9 to 230)	116.7 (101.6 to 133.8)	− 41.8 (− 48.9 to − 33.9)
**Rio Grande do Sul**	291,093 (263,932 to 317,669)	325,972 (295,523 to 355,685)	12 (6.8 to 17.3)	3137.2 (2844.5 to 3423.7)	2912 (2640 to 3177.5)	− 7.2 (− 11.5 to − 2.8)	4234.4 (3833.1 to 4621.4)	2168.8 (1963.7 to 2370.1)	− 48.8 (− 51 to − 46.5)	12,180 (11,013 to 13,282)	15,676 (14,127 to 17,231)	28.7 (23 to 34.7)	131.3 (118.7 to 143.1)	140 (126.2 to 153.9)	6.7 (1.9 to 11.7)	218.1 (192.9 to 241.7)	105.9 (95.1 to 116.4)	− 51.4 (− 53.7 to − 49)
**Santa Catarina**	109,782 (100,050 to 119,353)	159,102 (144,539 to 173,402)	44.9 (38.4 to 52.3)	2409.3 (2195.8 to 2619.4)	2287.8 (2078.4 to 2493.4)	− 5 (− 9.3 to − 0.2)	4160.4 (3776.4 to 4534)	2034.1 (1846.8 to 2215.3)	− 51.1 (− 53.3 to − 48.6)	4543 (4122 to 4957)	7275 (6573 to 7931)	60.1 (52.8 to 67.8)	99.7 (90.5 to 108.8)	104.6 (94.5 to 114)	4.9 (0.1 to 9.9)	221.5 (196.3 to 246.1)	103.4 (92.7 to 113.1)	− 53.3 (− 55.4 to − 50.9)
**Sergipe**	25,381 (22,627 to 27,933)	49,365 (44,971 to 53,849)	94.5 (83.4 to 106.1)	1677.5 (1495.5 to 1846.2)	2092.7 (1906.5 to 2282.8)	24.8 (17.7 to 32.2)	2911.8 (2603.1 to 3195.6)	2270.5 (2069.3 to 2474.5)	− 22 (− 26.3 to − 17.6)	1187 (1049 to 1313)	2219 (2015 to 2419)	86.8 (76.7 to 97.1)	78.5 (69.3 to 86.8)	94.1 (85.4 to 102.5)	19.8 (13.3 to 26.4)	145 (127.6 to 160.6)	107.9 (97.9 to 117.9)	− 25.6 (− 29.7 to − 21.5)
**São Paulo**	950,201 (856,603 to 1,038,673)	1,233,745 (1,118,989 to 1,345,216)	29.8 (24.3 to 36.1)	2956.8 (2665.5 to 3232.1)	2760.9 (2504.1 to 3010.3)	− 6.6 (− 10.6 to − 2.1)	4451.6 (4030.1 to 4839.1)	2335.2 (2113.1 to 2544.3)	− 47.5 (− 49.7 to − 45.2)	38,308 (34,776 to 41,763)	55,449 (50,418 to 60,463)	44.7 (38.8 to 50.9)	119.2 (108.2 to 130)	124.1 (112.8 to 135.3)	4.1 (− 0.1 to 8.5)	229.1 (201.2 to 254.7)	111.7 (101.4 to 122.3)	− 51.2 (− 53.4 to − 48.6)
**Tocantins**	11,812 (9806 to 13,863)	27,018 (23,822 to 30,329)	128.7 (103.7 to 163.1)	1287.5 (1068.8 to 1511)	1695.1 (1494.6 to 1902.8)	31.7 (17.2 to 51.4)	2682.8 (2277.1 to 3108.5)	1916.2 (1692.4 to 2145.3)	− 28.6 (− 35.6 to − 19.4)	446 (378 to 518)	1194 (1051 to 1347)	167.9 (139.7 to 201.2)	48.6 (41.2 to 56.5)	74.9 (65.9 to 84.5)	54.2 (37.9 to 73.4)	145.2 (123 to 168.7)	90.2 (79.3 to 101.8)	− 37.9 (− 44.7 to − 30.6)

**Fig. 4:**
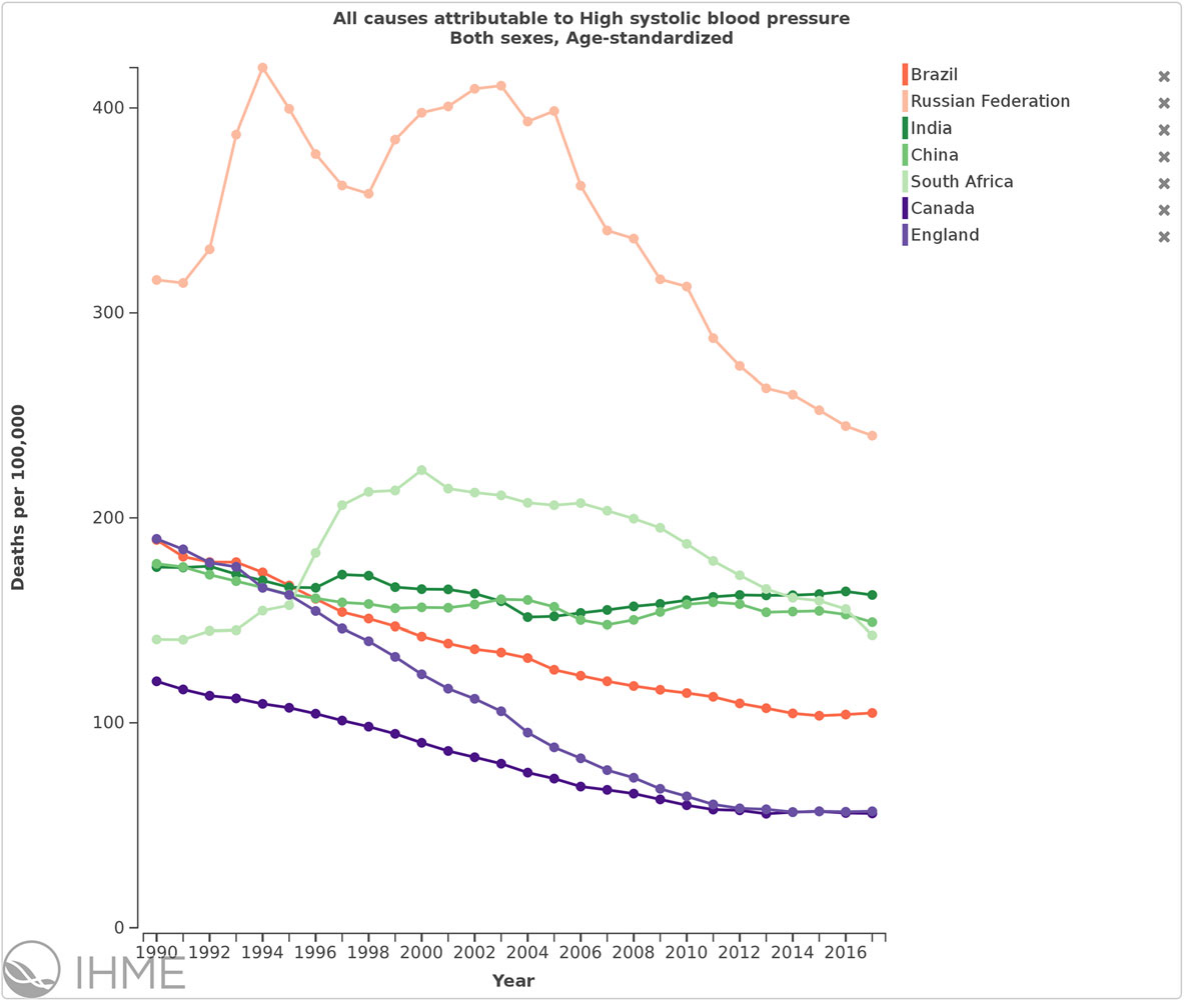
Trends in all causes of deaths attributable to high systolic blood pressure in Brazil, Russia, India, China, and South Africa (BRICS), Canada, and England, both sexes and age standardized, 1990–2017

When evaluating the age-standardized death rate attributable to HSBP in Brazil, it has fallen 44.6% (95% UI 42.9 to 45.9%), from 189.2 (95% UI 168.5 to 209.2) deaths to 104.8 (95% UI 94.9 to 114.4) deaths per 100,000 from 1990 to 2017. Comparing to other countries with similar development stage, such as other countries from the BRICS group (Brazil, Russia, India, China, and South Africa), Brazil has reduced the all-cause deaths attributed to HSBP from 2nd to the 5th and least position of the group from 1990 to 2017, although the rate of deaths attributed to HSBP in the country are still higher than other countries with universal healthcare, such as Canada and England, shown for comparison (Fig. 4).

Table 2 shows the all age deaths and DALYs, in 1990 and 2017, and percent change of deaths and age-standardized death rates, DALYs, and age-standardized DALY rates between 1990 and 2017 attributable to high blood pressure in Brazil and its FUs.

### Causes of deaths and DALYs attributable to HSBP

**Fig. 5:**
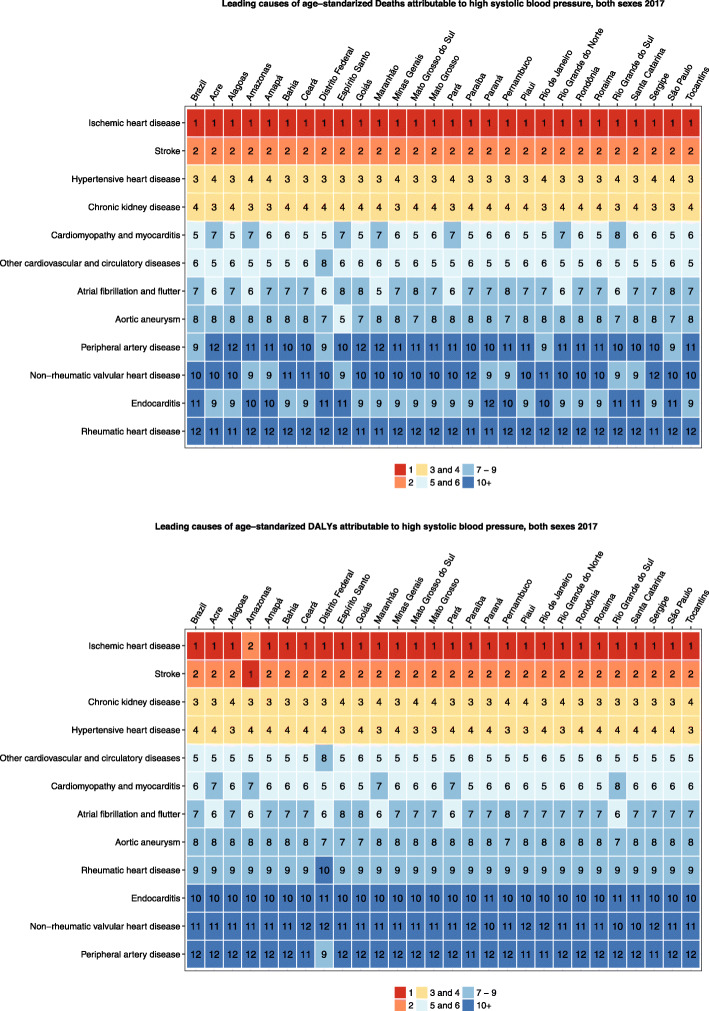
Main causes of deaths (**a**) and DALYs (**b**) attributable to high systolic blood pressure, both sexes, Brazilian federal units, 2017

Regarding the leading causes of death attributable to HSBP, ischemic heart disease is in the 1st position, followed by stroke (Additional file 2). While the deaths and DALY due to the main causes of death attributable to HSBP are decreasing, deaths due to peripheral artery disease, atrial fibrillation, and aortic aneurysm are rising. Figure 5 shows that they are similar across federal units. Hypertensive heart disease and chronic kidney disease alternate in the 3rd and 4th position depending on the federal unit.

**Fig. 6:**
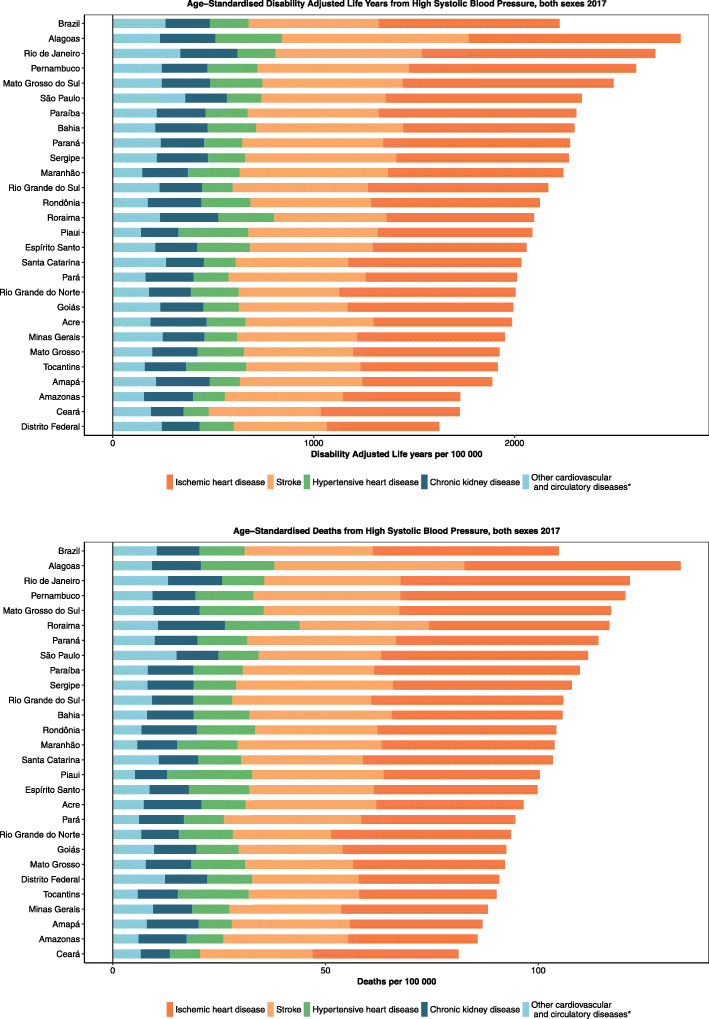
Age-standardized causes of **a** deaths and **b** DALYs attributable to high systolic blood pressure in Brazil and its Federal Units in 2017, both sexes, for men and women

Additional file 2 demonstrates all age deaths and DALYs in 1990 and 2017 and percent change of deaths and age-standardized death rates, DALYs, and age-standardized DALY attributable to HSBP, for cardiovascular diseases, for both sexes and stratified by sex, in Brazil. Figure 6 demonstrates the main causes of deaths and DALY attributable to HSBP across Brazilian federal units.

### Drivers of change in mortality and morbidity attributed to HSBP

**Fig. 7:**
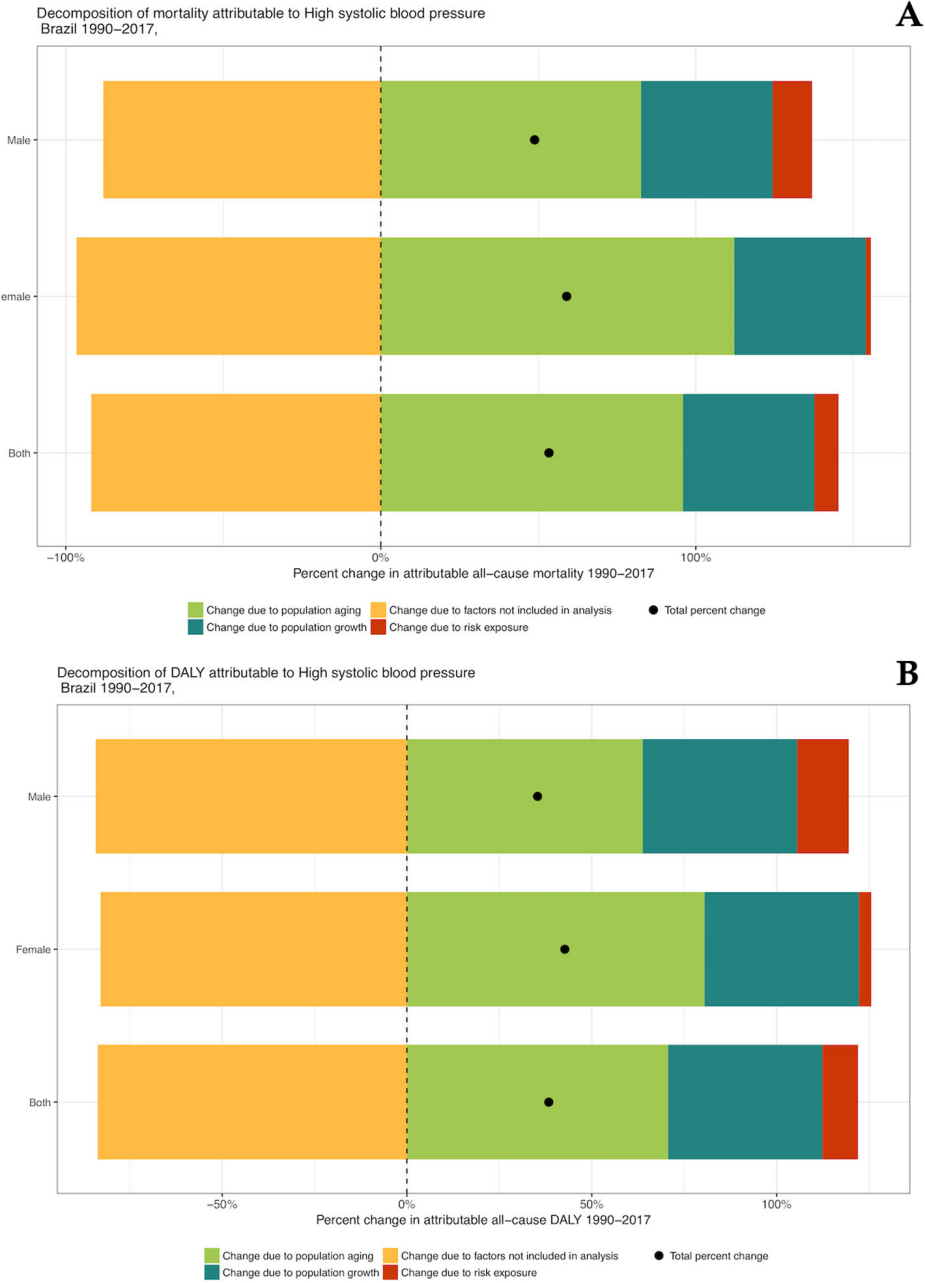
Percent change in deaths (**a**) and DALYs (**b**) attributable to high systolic blood pressure in Brazil, 1990–2016, due to population growth, population aging, trends in exposure included in GBD 2016, and all other (risk-deleted or residual) factors

Figure 7 reveals that the rise in deaths and DALY attributable to HSBP in Brazil was mainly due to population aging, particularly among women, followed by population growth. Changes due to risk exposure played a more important role in males. Changes in unmeasured factors (“not included in the analysis”)—including health care access and quality—contributed decreasing burden, but were offset by increases due to the other factors (population aging, growth, and risk exposure) (Fig. 7).

### Correlation of death and DALY rates attributable to HSBP to SDI

**Fig. 8:**
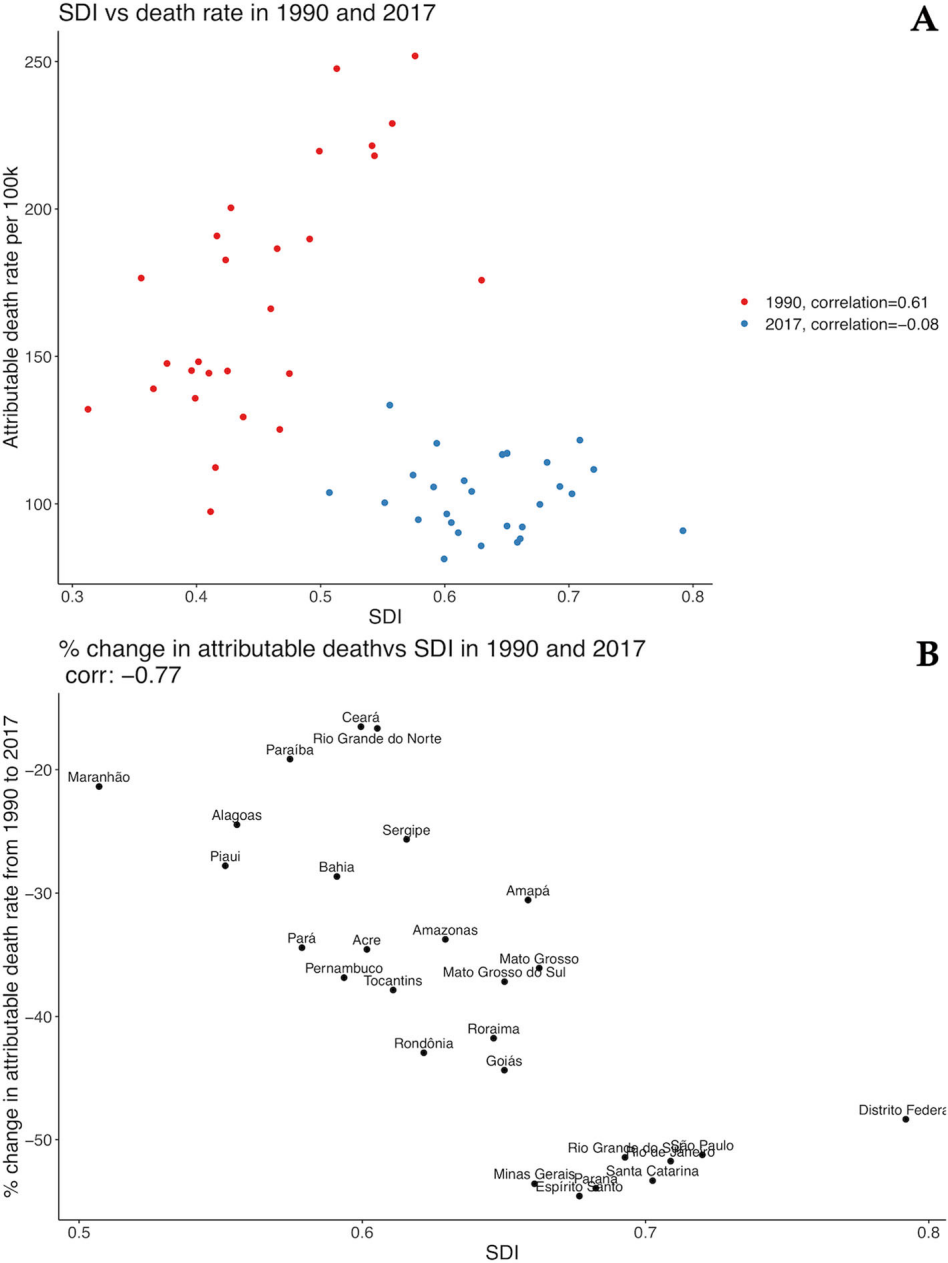
Correlation between age-standardized death rates attributable to high systolic blood pressure and the 2017 sociodemographic index (SDI) in 1990 and 2017 for both sexes, in Brazilian federal units (**a**) and percent change in death rates attributable to HSBP between 1990 and 2017 (**b**)

Figure 8a, b reveals that the reduction in the all-cause age-standardized death rates attributable to HSBP is heterogeneous across federal units. For example, in Ceará, the death rate reduced from 97.3 (95% UI 83.6 to 112.0) per 100,000 in 1990 to 81.3 (95% UI 71.7 to 90.5) per 100,000 in 2017 (16.5% [95% UI 8.9 to 23.3%] decrease), while in Espírito Santo the change was from 219.7 (95% UI 192.5 to 245.8) per 100,000 in 1990 to 99.8 (95% UI 90.4 to 109.2) per 100,000 in 2017 (54.6% [95% UI 51.9 to 57.1%] decrease). Figure 8a demonstrates the correlation between age-standardized death rates attributable to HSBP and the 2017 SDI. We found a moderate positive correlation (*r* = 0.61) in 1990 that was not maintained in 2017 (*r* = − 0.08). Figure 8b shows that the percent change in death rates attributable to HSBP between 1990 and 2017 was strongly and negatively correlated to the SDI in 2017 (*r* = − 0.77).

**Fig. 9:**
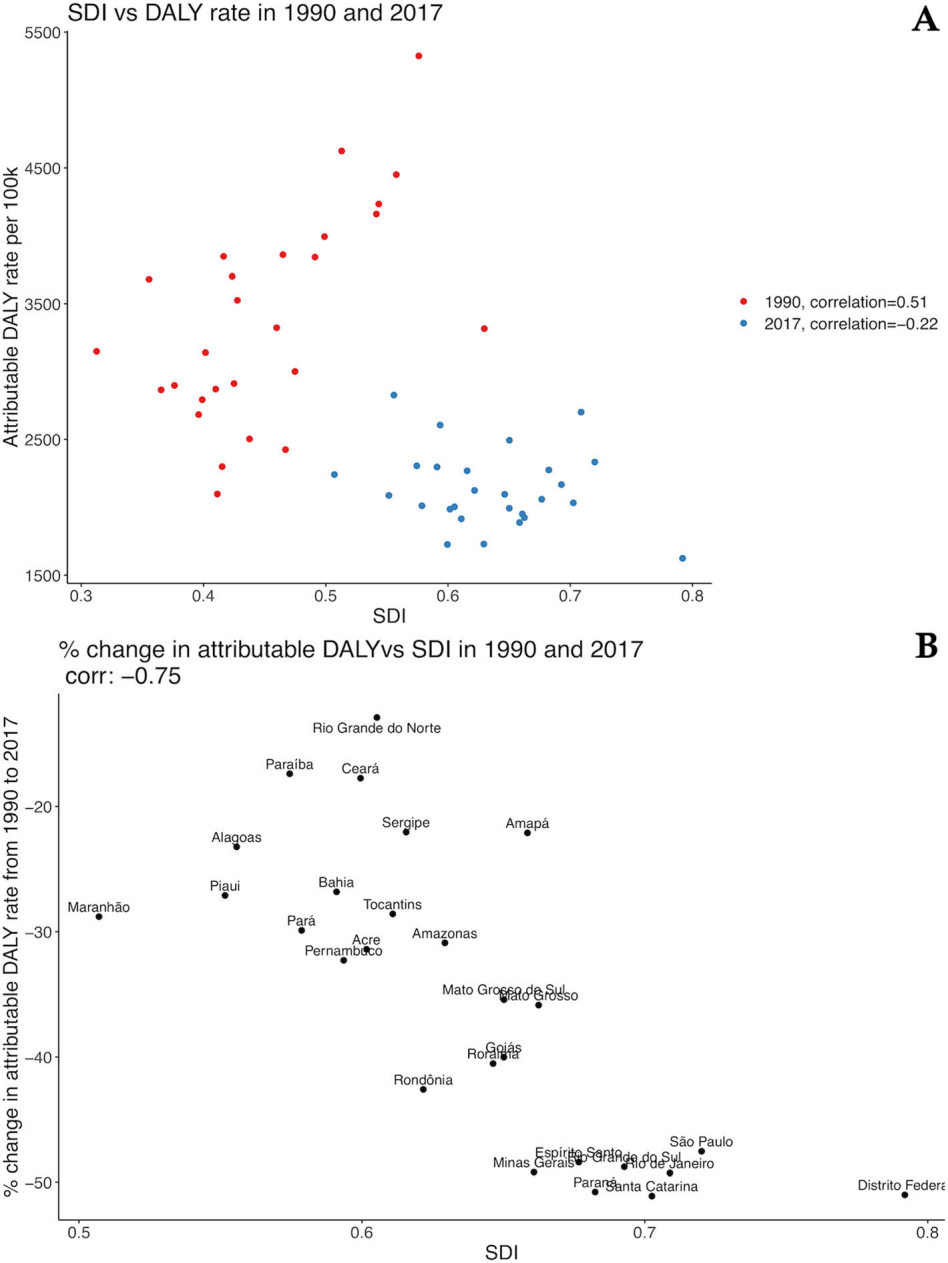
Correlation between age-standardized DALY rates attributable to high systolic blood pressure and sociodemographic index (SDI) in 1990 and 2017 for both sexes, in Brazilian federal units (**a**) and percent change in DALY rates attributable to HSBP between 1990 and 2017 (**b**)

Regarding age-standardized DALY rates attributable to HSBP, a similar pattern was observed. The moderate positive correlation found between it and SDI (*r* = 0.51) in 1990 was also not maintained in 2017 and became negative (*r* = − 0.22) (Fig. 9a), and the percent change in DALY rates attributable to HSBP between 1990 and 2017 was negatively correlated to the SDI (*r* = − 0.75) (Fig. 9b).

## Discussion

HTN is a risk factor for CVD, especially for cerebrovascular diseases, ischemic heart disease, and chronic kidney disease, and its recognition and control should be emphasized, in order to reduce the burden of its associated conditions. In our analysis, from 1990 to 2017, there was an annual (0.4%) increase—with an almost stable behavior in more recent years—in the age-standardized prevalence of HSBP, currently the leading risk factor for death in Brazil. Although the age-standardized death and DALY rates due to HSBP are declining in Brazil, possibly as a result of successful health policies, the total number of deaths and DALY are increasing mainly due to population aging. Moreover, the reduction in age-standardized death and DALY rates are heterogeneous across FU, being more pronounced in the more developed FU.

Several factors, including sociodemographic, ethnical, cultural, dietary, and behavioral issues, may account for the differences in the burden of HSBP between populations and also for the trends over decades. Global prevalence estimates may also vary according to the methodology applied. The pooled age-standardized prevalence of HSBP or high DBP in 200 countries was estimated at 24.1% (95% UI 21.4 to 27.1%) in males and 20.1% (95% UI 17.8 to 22.5%) in females in 2015, with the authors underscoring the decline observed in Latin America between the 1970s and 2015, although Brazil was not specifically mentioned [19]. In the GBD study, involving 195 countries, the estimated global prevalence of HSBP was 17.1% in 2017 [9, 11].

Several studies have been conducted on the prevalence of HTN in Brazilians cities and regions in the last decades, although there is substantial variability of definitions and methodology [1, 10]. Concerning country-wide studies, the Brazilian Health Ministry has been conducting, since 2006, a yearly telephonic survey in FU capitals for chronic diseases and risk factors, the VIGITEL, with a question about medical diagnosis of hypertension [20, 21]. More recently, in 2013, direct blood pressure measurements, as well as the information about the use of anti-hypertensive drugs, were incorporated [3]. The addition of these data to GBD was crucial, as it brings more realistic information regarding the profile of the population with HTN. The prevalence of HSBP by GBD estimates in Brazil, however, cannot be directly compared to previous studies, considering methodological particularities of the GBD model, in which prevalence is derived from a continuous distribution of systolic blood pressure (with a ≥ 140 mmHg threshold in this analysis), and patients with isolated diastolic HTN or those controlled by antihypertensives are not included [9]. This concern is even more compelling in regions with moderate to good access to medications, where patients under treatment with SBP levels below 140 mmHg will not meet the cutoff derived from the GBD continuous estimation of systolic BP. Most previous population-based studies [1, 8, 10] used as the diagnostic criteria the presence of blood pressure (BP) ≥ 140/90 mmHg or the use of BP lowering medication, which necessarily led to a higher prevalence than observed in GBD. Indeed, a meta-analysis from 2012 showed a mean prevalence of HTN of 28.7% (26.2–31.4%) for decade of 2000s [22], while the National Health Survey 2013 (adults > 18 years old) showed a general prevalence of 32.3% considering direct measurement and/or reported use of medication, contrasting with 21.4% for self-reporting and 22.8% for measured HTN [3]. For the burden estimates, the above concern is not applied, because HSBP was considered as exposure above a TMREL of 110–115 mmHg. However, for the purpose of estimating disease burden attributable to HSBP, the GBD method of estimating the full distribution of SBP regardless of medication use seems to be the best approach, as a continuous exposure measure takes into account the nuance of different risks for the different levels of BP.

Analyzing our results stratified by sex, it should be noted that the prevalence was overall higher among men during the study period, following a classical trend of HTN [13], despite the growing similarities of health behaviors between sexes in the past decades [23]. Besides biological determinants, such as estrogen protection, several other complex cultural, environmental, behavioral, and healthcare access factors may be implicated in this difference by sex. Higher prevalence of HSBP in men differs from the HTN prevalence estimated by self-reported population surveys [21] which tend to overestimate prevalence in women, whom are generally more adherent to medical services.

The small percent increase in age-standardized prevalence observed in Brazil—especially in men—was relatively homogeneous across the FUs. Of note, only in Rio Grande do Sul and Santa Catarina, there was no variation or even a slight reduction trend in this period, considering the UIs. In the VIGITEL telephone survey, the prevalence of self-reported medical diagnosis of HTN did not change from 2006 to 2017 [20]—but a non-significant increasing trend in the past years was observed—while a previous meta-analysis of population-based studies showed a trend toward decreasing prevalence from 1987 to 2007 [8]. Considering the limitation of all sources of data for evaluating this discordance, only a new National Health Survey, planned to be conducted in 2019, will be able to give a definite response for this question. However, other related risk factors, such as obesity [24] and diabetes [25], have already increased in prevalence during the same period, suggesting there is room for health promotion and prevention strategies that could lead to a better control of cardiovascular risk factors in Brazil. Also, considering the GBD methodology, HSBP can be understood as undetected or inadequately treated HTN, and the stable-to-growing trend observed in our analysis may be mainly linked to unsuccessful treatment. In this scenario, greater access to home measurement and better efficacy of self-management programs—including new technology for remote medical advice—may improve BP control.

Despite the observed slight growth of HSBP prevalence, the trends in the burden attributable to HSBP in Brazil, with decreasing age-standardized rates, suggest that efficient health policies for CVD control were implemented in the period. The increase in total numbers of deaths and DALY attributable to HSBP depict the effect of population aging and growth in the country. To analyze the drivers of the abovementioned trends is essential for health policy planning [26]. The relative contributions of population aging reflect life expectancy at birth that increased continuously from 1950 to 2017, to 72 years for men and 79 years for women. In Brazil, this was mainly due to the decline in under-5 mortality, with a still high mortality among young adult men driven by interpersonal violence [27]. The relatively small change due to risk exposure observed predominately in men could be attributed to the combined effect of birth cohorts’ improvements and treatment of cardiovascular and cardiometabolic diseases, which results in the decrease of CVD and increase in IHD [26]. This can be also interpreted as a tradeoff between worsening determinants of HTN—especially age—versus better healthcare. Conversely, the negative trend of “other causes” (not included in the analysis) is debatable and may also reflect healthcare and other unmeasured factors—as population awareness, etc. —or be a result of improving certainty around measurement in more recent years. A scenario mainly driven by the rapid change in age composition urges health systems to develop long-term action plans for improving primary prevention and sustainable patient-centered educational programs for healthy aging.

In comparison to other BRICS countries, the mortality rate per 100,000 inhabitants attributable to HSBP between 1990 and 2017 decreased 45% in Brazil and 24% in Russia, while it practically did not change in the other countries—with even an increasing trend in South Africa—over the period. This finding also suggests the relative efficiency of the large-scale health policies implemented in the country for HTN control, such as established follow-up protocols and multidisciplinary approach for HTN care in the primary health setting, along with free access to antihypertensives [6, 8]. However, among other countries with universal access to health such as Canada and England, we find attributable mortality rates to be about half those observed in Brazil in 2017. Notably, England and Brazil had comparable attributable mortality rates in 1990, indicating that even greater prevention of burden due to HTN may be possible in Brazil. The 2011 Brazilian plan to confront the non-communicable diseases (NCDs) [28], consonant with the World Health Organization (WHO) Action Plan [29] and the United Nations 2030 Sustainable Development Goals [30], emphasizes, among other approaches aiming a 25% reduction of NCD-associated mortality by 2025, the control of HTN and many of its determinants. However, considering the stable trend of prevalence and the absolute burden, the results are still suboptimal. One of the reasons for this may be that greater healthcare, medication access, and public policies to prevent CVD are being counterbalanced by unsuccessful approaches to control the determinants of HTN, such as overweight, alcohol intake, and physical inactivity.

It is interesting that prevalence varied considerably among FUs, which may be partially explained by different stages of the epidemiological transition across the country. Age-standardized death and DALY rates were higher in FUs with higher SDI in 1990, and the greatest reduction in burden was observed in these locations. In fact, considering the SDI as a proxy for socioeconomic development, the strong positive correlation observed in 1990 contrasts with the absence of significant correlation in 2017, revealing that greater sociodemographic development was associated with the reduction in the burden attributable to HSBP in Brazil. This is supported by the strong negative correlation between the percent changes in death and DALY rates attributable to HSBP between 1990 and 2017 and the 2017 SDI. The relation of socioeconomic development and disease burden in Brazil has already been observed: significantly negative correlation coefficients between the municipal HDI (an index that similarly reflects local development) in 2000 and 2013 and mortality rates for CVD and hypertensive diseases in the state of Rio de Janeiro have been reported [31]. Higher socioeconomic development may be associated with greater awareness about the effects of HTN and better access to healthcare, such as public and private programs related to education, diet, behaviors, and risk factors’ control over the period evaluated. Moreover, the improvement in socioeconomic conditions of the Brazilian FUs—while in 1990 the lowest SDI was 0.31 in Maranhão, in 2017 it was 0.51—and the consequent reduction in socioeconomic gap between FU in Brazil from 1990 and 2017 may have contributed to reduce the magnitude of the statistical correlation with disease burden. According to the United Nations Organizations definition, about 50% of the Brazilian FU reached a high Human Development Index (HDI) (≥ 0.7) by the 2010 decade, explaining the decreasing power of this association [32] and highlighting the health burden posed by socioeconomic disparities. In Brazilian case, the reduction of the HTN burden clearly followed social development. These observations follow a global trend of linear increase of HSBP burden and SDI in places with low baseline SDI [9], hypothetically as a result of lifestyle/behavioral changes increasing metabolic risk factors as the country develops. A drop of HSBP is then observed at high SDIs, when development is enough to allow for adequate BP control.

HSBP accounted for the highest proportion of deaths in 2017 in both sexes, followed by dietary risks and increased body mass index in women, with tobacco consumption prevailing over dietary risks for men. Regarding the leading causes of DALY, the overall pattern substantially changed as a reflex of the epidemiological transition, from child and maternal malnutrition in 1990 (with HSPB in the 4th position) to tobacco in 2017, followed by HSBP. These findings differ from those reported in 2015, when dietary risks—improved by successful strategies focused on diet and other health behaviors—were the main cause of DALYs [33]. On the other hand, a recent study observed a decrease in the smoking reduction trend, which varied from − 23.4% in the years 2010–2014 to − 2.9% in the period 2015–2017, even with an increase in prevalence in the population with ≥ 9 years of schooling—an alarming trend, considering the successful anti-tobacco initiatives in the past decades. The authors point to one of the possible determinants: the fiscal austerity and the economic crisis that Brazil is currently experiencing [2], limiting the expansion of health education programs. These data point toward the need for individualized approaches for men and women in prevention and awareness programs, reinforcing—as examples—tobacco cessation for men, while focusing on dietary habits and obesity for women. HTN, for instance, must remain as a top priority for policy-making.

Ischemic heart disease and stroke remained as the first and second leading causes of age-standardized deaths and DALYs attributable to HSBP respectively, in both sexes, in 2017, in Brazil and its FU [34]. This suggests a more chronic end-organ damage profile, also deeply associated with other common risk factors such as tobacco, diabetes, and cardiometabolic factors [35]. Similar to aging, the growing impact of such factors—affecting ischemic diseases—can be understood as a product of the late epidemiological transition. Also, better access to healthcare seems to result in a more acute reduction of morbidity and mortality associated with kidney disease and hypertensive heart disease [13]. For the reduction of this burden, there has been much debate about adequate targets for blood pressure control and their clinical feasibility, as meta-analyses and primary data strongly suggest cardiovascular mortality benefits for a target SBP < 120 mm Hg [13, 36, 37]. These observations support the assumption that adequate SBP control modifies the associated mortality in a progressive fashion, although the precise subpopulations that benefit from intensive control and the optimal targets remain unclear [13]. The new BP target recommendations [35] have not yet been incorporated by Brazilian guidelines [4]. However, the ability to control determinants of HTN (e.g., diet, salt and alcohol intake, obesity) in the population may be more important than the unclear benefits of intensified BP targets.

Thus, cardiovascular health overall improved substantially in Brazil from 1990 to 2017, with important differences across the geographical regions of the country [38]. This reduction was more pronounced in the states in the southern and southeastern regions possibly being influenced by socioeconomic development [39, 40]. Our findings from the GBD 2017 estimates reinforce these observations, pointing toward the need for a broad discussion about the directions of cardiovascular care in Brazil in the coming years. More than access to medical appointments, adequate long-term HTN control is highly dependent on population education to improve awareness and health promotion through a multidisciplinary approach, as well as improvement of the quality of care [6]. In part, this is the scope of the Family Health program that has been developed in the Brazilian primary care system since the 1990s, which should be further promoted [8]. Although it is necessary to provide universal access and high-quality healthcare, it is mandatory to also act on social determinants of health to sustainably reduce the burden of CVD [41].

### Limitations and strengths

The limitations of the GBD study models have been previously detailed [9]. Despite the improvement in availability of primary data in Brazil from 1990 to 2017, the publications are still heterogeneous, and data are still scarce for some regions, especially the less resourced ones as the North and Northeast. However, the implementation of health surveys in the country with measured blood pressures, such as the “National Health Survey” (*Pesquisa Nacional de Saúde—*PNS) are recently improving the knowledge of the epidemiological profile of the population [3]. Furthermore, 140 mmHg was the threshold for prevalence estimates, despite the changes in the 2017 American Heart Association/American College of Cardiology Guidelines driven by robust data [35, 36]. The past threshold, however, remains acceptable for a considerable proportion of the population. Also, of note, controlled HTN and isolated DBP were not considered for the estimates, although the latter is uncommon and unlikely to explain differences in prevalence. Although it is recognized that systolic values have stronger associations with health outcomes, these may also be sources of bias [13] accounting, along with abovementioned particularities of the GBD model, for the considerable lower prevalence of HSBP in comparison with primary HTN data. This precludes the direct comparison with other studies that used definitions that considered DBP and treated HTN, as mentioned, as different conditions are being measured. Finally, disability weights were not country-specific and may be prone to some imprecision, although some data has shown that they are relatively stable across different populations [14].

Despite the above limitations, GBD is a robust and broad epidemiological initiative, for estimating the morbidity and mortality due to HSBP in the entire Brazilian territory, especially in regions where primary data is scarce, where temporal-spatial complex models provide reliable estimates, previously unavailable [9, 13]. The lack of precision of some aspects of the subnational models in Brazil—requiring a close evaluation of uncertainty intervals provided by GBD—does not affect the main findings and the contribution of this approach to evaluate the health impact of HSBP and help develop policies for its confrontation. The main strength of this study is to be, at the best of our knowledge, the most comprehensive countrywide data demonstrating the significant reduction of age-standardized death and DALY rates attributable to HSBP in Brazil, but also the maintenance of HSBP as the main risk factor for death in the country.

## Conclusion

The age-standardized death rates attributable to HSBP are decreasing in Brazil, probably revealing the results of successful public health policies for CVD secondary prevention and control, markedly public campaigns and the availability of drug therapy in the public health system. However, prevalence data still shows a trend of increase that deserves to be confirmed by a new National Health Survey. Moreover, HSBP continues to be the main risk factor for death in the country and an increase in the total number of diseases attributable to HSBP in the near future is foreseen, mainly due to population aging and growth. These findings, along with the correlation between high SDI and the reduction in the burden attributable to HSBP, suggest that health policies must focus on healthy aging and the underserved population, emphasizing specific strategies for HTN screening, treatment, and adherence. Sensitization at different levels, from health policy-makers to civil society organizations, is crucial for the development of contemporary strategies to confront HSBP in Brazil in order to diminish the burden of health loss due to HSBP in the next decades.

## Supplementary information

**Additional file 1.** Global standard population distribution utilized by the Global Burden of Disease 2017 study.

**Additional file 2: Table S3.** All age deaths and DALYs in 1990 and 2017 and percent change of deaths and age-standardized death rates, DALYs, and age-standardized DALY attributable to high blood pressure, for cardiovascular diseases (total) and for each level 2 cardiovascular disease for both sexes (A), men (B), and women (C), in Brazil.

## Data Availability

The datasets used and/or analyzed during the current study are available from the corresponding author on reasonable request. Data we used in this article are publicly available online on the official website of Institute of Health Metrics and Evaluation (http://ghdx.healthdata.org/gbd-results-tool).

## Abbreviations

BP: Blood pressure
BRICS: Brazil, Russia, India, China, and South Africa
CRA: Comparative Risk Analysis
CVD: Cardiovascular disease
DALY: Disability-adjusted life years
DBP: Diastolic blood pressure
FU: Federal units
GBD 2017: Global Burden of Disease 2017 study
HSBP: High systolic blood pressure
HTN: Hypertension
PAF: Population-Attributable Fraction
RR: Relative risks
SDI: Sociodemographic index
SUS: *Sistema Único de Saúde*
TMREL: Theoretical minimum risk exposure level
VIGITEL: *Vigilância de Fatores de Risco e de Proteção para Doenças Crônicas*
YLD: Years lived with disability
YLL: Years of life lost
95% UI: 95% uncertainty intervals
